# FBLN7 mediates vascular smooth muscle cell phenotype switching and vascular remodeling in hypertension

**DOI:** 10.7150/thno.102593

**Published:** 2024-11-04

**Authors:** Guoqing Yao, Xuehui Zheng, Yang Hu, Yuan Zhao, Binghui Kong, Yun Ti, Pei li Bu

**Affiliations:** State Key Laboratory for Innovation and Transformation of Luobing Theory; Key Laboratory of Cardiovascular Remodeling and Function Research, Chinese Ministry of Education, Chinese National Health Commission and Chinese Academy of Medical Sciences; Department of Cardiology, Qilu Hospital of Shandong University, Jinan, 250012, China

**Keywords:** Vascular remodeling, FBLN7, Phenotypic transformation, Hypertension, Syndecan

## Abstract

**Rationale:** Arterial remodeling serves as a pivotal mechanism underlying the development of diseases such as hypertension. Fibulin-7 (FBLN7), an adhesion protein, remains enigmatic regarding its role in these pathological processes. This study aims to explore whether FBLN7 influences vascular remodeling and its underlying mechanisms.

**Methods:** We generated FBLN7 knockout mice and smooth muscle-specific FBLN7 overexpression mice. Vascular remodeling models were established by administering angiotensin II (Ang II) for 28 days. RNA sequencing, western blot, and immunofluorescence assays were employed to investigate the biological function of FBLN7 in vascular smooth muscle cells (VSMCs). The interaction mechanism between FBLN7 and cell membrane receptors was explored through mass spectrometry analysis, co-immunoprecipitation techniques and molecular dynamics simulations.

**Results:** Bioinformatics analysis revealed an upregulation of FBLN7 expression in the vascular remodeling model, with FBLN7 predominantly localized in VSMCs. Subsequent *in vivo* validation demonstrated that FBLN7 knockout attenuated Ang II-induced vascular remodeling, reducing aortic wall thickness and collagen formation. Conversely, VSMC-specific overexpression of FBLN7 via AAV vectors exacerbating the remodeling phenotype. Functionally speaking, FBLN7 potentiates Ang II-mediated phenotypic transformation. Mechanistically, FBLN7 interacts with the extracellular and transmembrane domains of syndecan-4 (SDC4) via its C-terminal region, affecting SDC4 signaling and dimer formation. This interaction inhibits SDC4-mediated activation of the Rho-associated protein kinase pathway, subsequently reducing nuclear translocation of myocardin-related transcription factor A, leading to decreased transcription of genes associated with the contractile VSMCs phenotype.

**Conclusions:** These findings reveal FBLN7 promotes the transition of VSMCs from a contractile to a synthetic phenotype, thereby aggravating vascular remodeling. This provides further insights into the pathogenesis of vascular remodeling and potential therapeutic strategies.

## Introduction

Vascular remodeling serves as the primary pathological hallmark of numerous chronic vascular diseases and represents a significant risk factor for cardiovascular complications, posing a grave threat to human health. This process not only highlights the underlying pathology but also underscores the urgent need for effective interventions to mitigate its detrimental effects 1, 2. Initially, vascular remodeling serves as an adaptive measure, bolstering the vessel's resilience against pressure and inflammation-mediated damage. However, as the disease progresses, this process can become irreversible and pathogenic 3-5. Consequently, understanding the etiology and pathogenesis of vascular remodeling is crucial for developing efficacious treatment strategies aimed at halting or mitigating its progression.

The aorta is anatomically structured by a complex interplay of intimal endothelial cells, medial vascular smooth muscle cells (VSMCs), and adventitial fibroblasts. Notably, VSMCs within the media of the aorta exhibit remarkable plasticity, functioning as contractile differentiated cells 6-8. These cells possess the ability to transition between a contractile phenotype and a dedifferentiated, synthetic phenotype 9, 10. This transformation is characterized by the loss of markers typical of the contractile phenotype, such as α-smooth muscle actin (α-SMA), calponin-1 (CNN1), and smooth muscle 22α (SM22α), coupled with an upregulation of extracellular matrix components 11, 12. Ultimately, this pathological transformation leads to thickening of the vascular wall, luminal narrowing, and other adverse alterations 13, 14.

Fibulin-7 (FBLN7), a matricellular protein, was first identified by Susana de Vega *et al.* through differential hybridization using a mouse tooth germ cDNA microarray 15. Certain members of the fibulin family, such as FBLN4 and FBLN5, are implicated in the development of vascular elasticity 16, 17. Unlike these members, FBLN7 seems to exhibit somewhat different functions, sparking curiosity and exploration into its distinct functional roles 18. Since its identification, several reports have highlighted the regulatory role of the C-terminal fragment of the FBLN7 protein (FBLN7-C) on the functions of human umbilical vein endothelial cells (HUVECs) and leukocytes 19-21. FBLN7-C can bind to various cell surface receptors, including integrins and heparan sulfate proteoglycans (HSPGs), although the specific mechanisms of interaction between FBLN7 and these receptors remain unclear 22. Recent research suggested that FBLN7 is involved in the pathogenesis of myocardial infarction, with FBLN7 knockout mice exhibiting reduced myocardial fibrosis post-infarction 23. However, the role of FBLN7 in other cardiovascular diseases remains largely unexplored.

In this study, we discovered that FBLN7 serves as a crucial regulator of VSMCs phenotypic transformation and participates in the progression of hypertensive vascular remodeling. FBLN7 can bind to the extracellular and transmembrane domains of the cell surface syndecan-4 (SDC4) through its C-terminus, thereby influencing SDC4 function and dimer formation. This interaction leads to the inhibition of the Rho-associated protein kinase (ROCK)/myocardin-related transcription factor A (MRTF-A) signaling pathway, promoting the transition of smooth muscle cells from a contractile phenotype to a synthetic phenotype and exacerbating vascular remodeling.

## Results

### FBLN7 in VSMCs is associated with vascular remodeling

**Figure 1:**
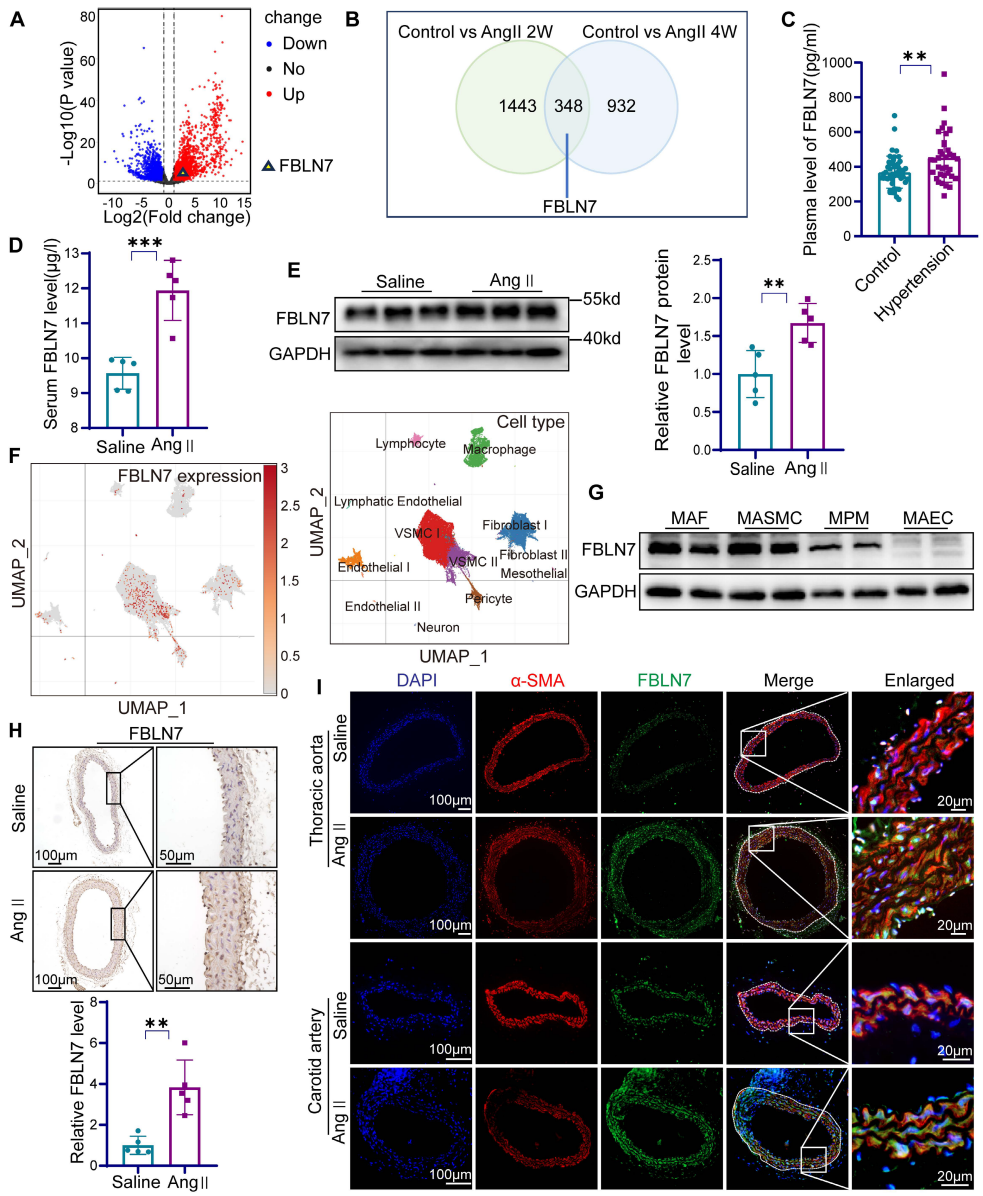
** The expression of FBLN7 is increased in vascular remodeling.** (A) Volcano plot analysis from GEO database (GSE 269845, Control = 5, AAA = 4) provides evidence of differential FBLN7 expression (P < 0.05). (B) Venn diagram analysis from GEO database (GSE 175588, Control n = 2, angiotensin II (Ang II) 2w n = 2, Ang II 4w n = 2) provides evidence of differential FBLN7 expression. (C) Elisa analysis of FBLN7 in peripheral blood from hypertension patients (n = 35) and normal controls (n = 47). (D) Elisa analysis of FBLN7 in mouse peripheral blood after 4 weeks of Ang II or saline infusion (n = 5). (E) Western blot analysis and the relative quantification of the expression of FBLN7 in the aorta of mice after 4 weeks of Ang II or saline infusion (n = 5). (F) Uniform manifold approximation and projection (UMAP) revealed the distribution of FBLN7 within single-cell clusters of the human aorta. (G) Western blot analysis of FBLN7 expression levels in mouse aortic fibroblasts (MAFs), mouse aortic smooth muscle cells (MASMCs), mouse peritoneal macrophages (MPMs), and mouse aortic endothelial cells (MAECs). (H) Representative images and the relative quantification of FBLN7 immunohistochemical staining in aortic tissue after 4 weeks of infusion with Ang II or saline infusion (n = 5). (I) Representative immunofluorescence images of α smooth muscle actin (α-SMA, red) and FBLN7 (green) in mouse thoracic aorta and carotid artery after 4 weeks of Ang II or saline infusion. Data were presented as mean ± SEM. Statistical significance was assessed using unpaired two-tailed Student's t-test. **p < 0.01, ***p < 0.001.

To preliminarily investigate whether FBLN7 is involved in vascular remodeling, we analyzed high-throughput sequencing data from two typical vascular remodeling models, abdominal aortic aneurysm (AAA) and hypertensive vascular remodeling, in the GEO database. In dataset GSE 269845, a volcano plot revealed significantly elevated FBLN7 expression in the AAA patient group (n = 4) compared to the control group (n = 5) (|fold change| > 1.5, P = 1.86E-04) **(Figure 1A)**. Another dataset from aortic tissues of mice subjected to Ang II-induced hypertensive vascular remodeling, with groups consisting of controls (n = 2), a 2-week Ang II infusion group (n = 2), a 4-week Ang II infusion group (n = 2), and a 5-week Ang II infusion group (n = 2). The results showed elevated FBLN7 expression at 2and 4 weeks post-Ang II infusion compared to controls **(Figure 1B)**.

FBLN7 is a secreted protein detectable in peripheral blood. We first measured FBLN7 levels in the peripheral blood of hypertensive patients and healthy controls, with the baseline data presented in the supplemental materials **(Table S1)**. The results showed significantly elevated systolic and diastolic blood pressure in hypertensive patients, along with a marked increase in plasma FBLN7 levels compared to healthy controls **(Figure 1C, Table S1)**. To validate these findings *in vivo*, we established a classic hypertension-induced vascular remodeling model by subcutaneously implanting Ang II pumps in 10-week-old C57BL/6 mice for 4 weeks. The FBLN7 levels in the peripheral blood of these mice were significantly increased in the Ang II-infused group** (Figure 1D)**. Western blot confirmed increased FBLN7 expression in the aorta following Ang II pump implantation **(Figure 1E)**.

Next, we explored FBLN7 expression in vascular wall cells. We analyzed the single-cell sequencing data of human aortic tissues conducted by James P. Pirruccello and colleagues in 2022 24. The data revealed that FBLN7 is expressed in various cell types, with significant expression observed in smooth muscle cells, fibroblasts, and pericytes **(Figure 1F)**.

Western blot analysis confirmed FBLN7 expression in mouse aortic smooth muscle cells (MASMCs), mouse aortic endothelial cells (MAECs), mouse aortic fibroblasts (MAFs), and mouse peritoneal macrophages (MPMs), with particularly high expression in MASMCs **(Figure 1G)**. Given that VSMCs are the most abundant cell type in the vascular wall and one of the most crucial cell types involved in vascular remodeling 25, we investigated the response of MASMCs to Ang II stimulation. The results demonstrated an increase in FBLN7 expression in MASMCs following the addition of Ang II **(Figure S1)**. Immunohistochemical staining of the thoracic aorta showed elevated FBLN7 expression within the aortic media **(Figure 1H)**. Immunofluorescence staining of the thoracic aorta and carotid artery indicated significantly increased FBLN7 expression following Ang II infusion, colocalizing with the smooth muscle cell marker α-SMA** (Figure 1I)**. These findings suggest that FBLN7 plays a critical role in hypertension-induced vascular remodeling, and it is of great significance to explore the role of FBLN7 in VSMCs during the process of vascular remodeling.

### FBLN7 knockdown alleviates Ang II-induced vascular remodeling

**Figure 2:**
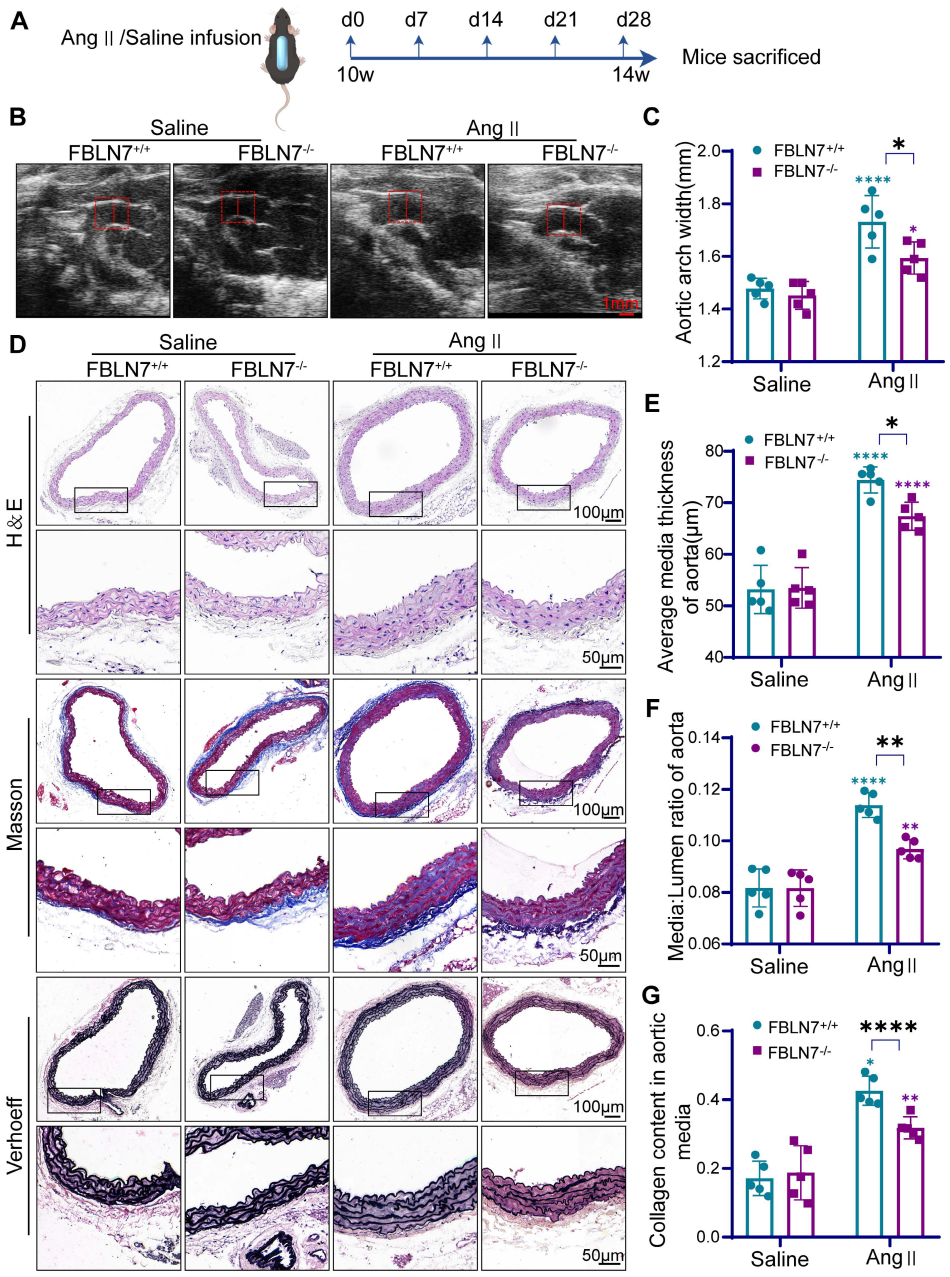
** The deficiency of FBLN7 in mice alleviates vascular remodeling.** (A) Timeline of mouse model construction. (B) Representative ultrasound images of thoracic aortas from wild type (FBLN7^+/+^) and FBLN7 knock out (FBLN7^-/-^) mice infused with Ang II or saline (n = 5). (C) Maximum aortic diameter measured under ultrasound (n = 5). (D-G) Vascular remodeling was analyzed in thoracic aortic sections from FBLN7^+/+^ and FBLN7^-/-^ mice infused with Ang II or saline (n = 5). Representative macro images of aortic sections stained with hematoxylin & eosin (H&E), Masson's trichrome (Masson), and Elastin-Verhoeff (EVG). The vascular media thickness (E) and ratio of aortic media to lumen (F) were calculated based on H&E staining, and the staining density of collagen (G) in the aortic wall was quantitatively analyzed using Masson staining. Data are presented as mean ± SEM. Statistical significance was assessed using two-way ANOVA with Bonferroni post hoc analysis. Green asterisk, versus FBLN7^+/+^-Saline group. Purple asterisk, versus FBLN7^-/-^-Saline group. *p < 0.05, **p < 0.01, and ****p < 0.0001.

To further investigate the role of FBLN7 in vascular remodeling, we generated FBLN7-deficient mice (FBLN7^-/-^) **(Figure S2A-B)**. No developmental abnormalities were observed in the FBLN7^-/-^ mice. Osmotic pumps containing Ang II or saline were then subcutaneously implanted in FBLN7^-/-^ mice and their wild-type (FBLN7^+/+^) littermates for 28 days **(Figure 2A)**. Tail blood pressure was measured on days 0, 7, 14, and 28, and vascular ultrasound of the aorta was performed at the beginning and end of the experiment. Results showed that arterial blood pressure significantly increased in both FBLN7^-/-^ and FBLN7^+/+^ groups after Ang II infusion compared to the saline group, with no significant difference between the two groups **(Figure S2C)**. Vascular ultrasound of the vessels indicated that FBLN7 knockout alleviated Ang II-induced aortic dilation compared to the control group **(Figure 2B-C)**.

To evaluate the impact of FBLN7 deletion on Ang II-induced vascular remodeling, hematoxylin & eosin (H&E), Masson's trichrome (Masson), and Elastin-Verhoeff (EVG) staining were performed to assess medial thickness, medial-to-lumen area ratio, and elastic fiber and collagen expression in the aorta. Compared to FBLN7^+/+^ mice, FBLN7^-/-^ mice exhibited significantly reduced medial thickness and a lower medial-to-lumen area ratio following Ang II infusion. Moreover, FBLN7 deficiency reduced Ang II-induced collagen deposition and restored elastin expression** (Figure 2D-G)**.

### VSMC-specific FBLN7 overexpression aggravates Ang II-induced vascular remodeling

**Figure 3:**
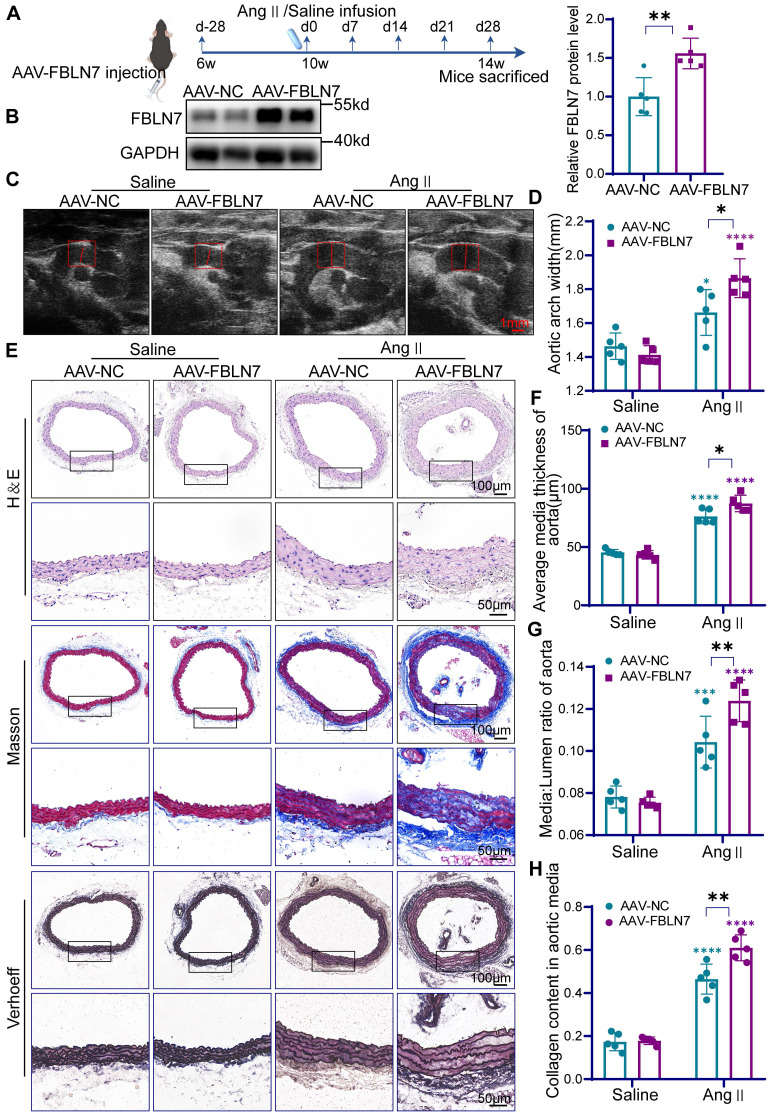
** Specific overexpression of FBLN7 in mouse VSMCs exacerbates vascular remodeling.** (A) Timeline for the mouse model construction. (B) Western Blot analysis and the relative quantification of FBLN7 overexpression efficiency in mice (n = 5). (C) Representative ultrasound images of the thoracic aortas from AAV-NC and AAV-FBLN7 mice after 4 weeks of saline or Ang II infusion. (D) Maximum aortic diameter measured under ultrasound (n = 5). (E-H) Analysis of vascular remodeling in thoracic aortic sections from AAV-NC and AAV-FBLN7 mice after 4 weeks of saline or Ang II infusion (n = 5). Representative macro images of aortic sections stained with H&E, Masson, and EVG. The vascular media thickness and ratio of aortic media to lumen (F, G) were calculated based on H&E staining, and the staining density of collagen (H) in the aortic smooth muscle wall was quantitatively analyzed using Masson staining. Data are presented as mean ± SEM. Statistical significance was assessed using unpaired two-tailed Student's t-test (B) or two-way ANOVA with Bonferroni post hoc analysis (D, F, G and H). Green asterisk, versus AAV-NC-Saline group. Purple asterisk, versus AAV-FBLN7-Saline group. *p < 0.05, **p < 0.01, ***p < 0.001 and ****p < 0.0001.

To further validate the role of FBLN7 in VSMCs during vascular remodeling, we overexpressed FBLN7 specifically in mouse VSMCs. Using tail vein injection of an adeno-associated virus (AAV9) vector driven by the SM22α promoter, we introduced FBLN7 (AAV-FBLN7) or a Flag-tagged empty vector (AAV-NC) targeting VSMCs in mice **(Figure 3A)**. The efficiency of FBLN7 overexpression was confirmed via immunohistochemistry, immunofluorescence, and western blotting **(Figure 3B, Figure S2D-E)**. As expected, AAV-FBLN7 significantly increased FBLN7 expression levels **(Figure 3B)**. Co-staining for the Flag-tag and α-SMA confirmed the VSMC-specific expression of FBLN7 in mouse aortic tissues **(Figure S2D)**. Overexpression of FBLN7 did not alter baseline blood pressure, and there was no significant difference in blood pressure between mice injected with AAV-FBLN7 and AAV-NC after 4 weeks of Ang II treatment **(Figure S2F)**.

In mice injected with AAV, Ang II infusion induced significant vascular remodeling, characterized by increased medial thickness, medial-to-lumen area ratio, and collagen deposition in the thoracic aorta **(Figure 3C-H)**. Moreover, AAV-FBLN7 treatment exacerbated these pathological changes** (Figure 3C-H)**. These results indicate that AAV-mediated overexpression of FBLN7 aggravates Ang II-induced vascular remodeling.

### FBLN7 modulates the phenotypic transformation of VSMCs

**Figure 4:**
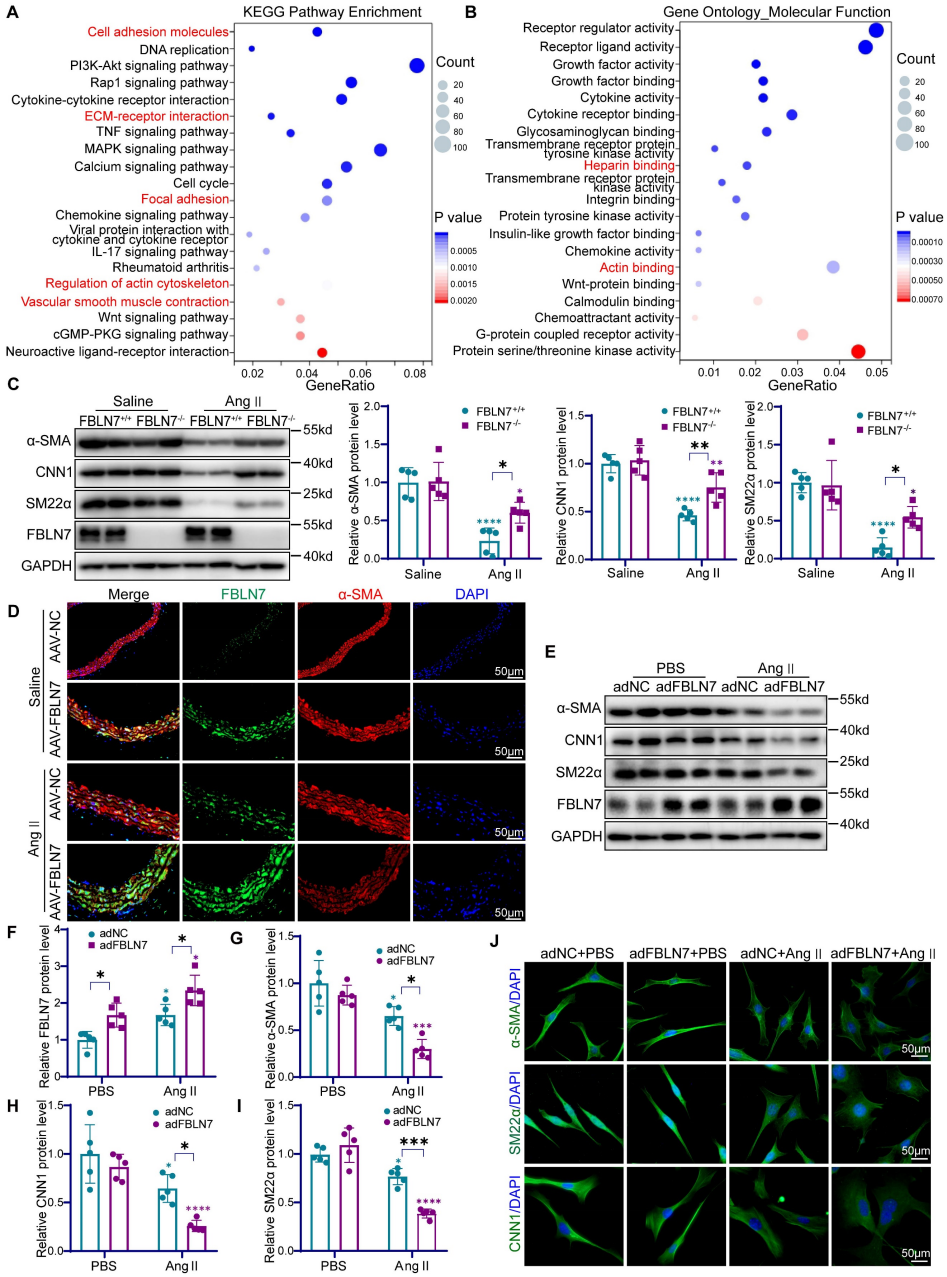
** FBLN7 leads to the loss of the contractile phenotype in VSMCs.** (A) Kyoto encyclopedia of genes and genomes (KEGG) pathway enrichment analysis of differentially expressed genes in FBLN7^+/+^ and FBLN7^-/-^ VSMCs following Ang II treatment (n = 3). (B) Gene ontology (GO) molecular function enrichment analysis of differentially expressed genes in FBLN7^+/+^ and FBLN7^-/-^ VSMCs following Ang II treatment (n = 3). (C) Western blot analysis and the relative quantification of α-SMA, CNN1, and SM22α expression in aorta tissue of FBLN7^+/+^ and FBLN7^-/-^ mice after infusion of saline or Ang II for 4 weeks (n = 5). (D) Representative images of immunofluorescence staining of α-SMA (red) and FBLN7 (green) in AAV-NC and AAV-FBLN7 mouse aorta tissue after infusion of saline or Ang II for 4 weeks. (E-I) Western blot analysis and the relative quantification of α-SMA, CNN1, and SM22α in adNC and adFBLN7 VSMCs treated with PBS or Ang II (n = 5). (J) Cellular immunofluorescence analysis of VSMC contractile markers (including α-SMA, CNN1, and SM22α) in adNC and adFBLN7 VSMCs treated with PBS or Ang II. Data are presented as mean ± SEM. Statistical significance was assessed using two-way ANOVA with Bonferroni post hoc analysis. Green asterisk, versus FBLN7^+/+^-Saline group (C); versus adNC-PBS group (F-I). Purple asterisk, versus FBLN7^-/-^-Saline group (C); versus adFBLN7-PBS group (F-I). *p < 0.05, **p < 0.01, ***p < 0.001 and ****p < 0.0001.

To explore how FBLN7 affects the function of VSMCs, we performed bulk RNA sequencing on Ang II-treated FBLN7^+/+^ and FBLN7^-/-^ MASMCs. Enrichment analysis revealed that the major differences between the two groups were associated with cell adhesion, cell membrane receptor binding, vascular smooth muscle contraction function, and actin cytoskeleton regulation **(Figure 4A-B, Table S2-3)**. Moreover, the expression of VSMCs contraction markers such as α-SMA, CNN1, and SM22α was elevated in FBLN7 knockout cells **(Figure S3A)**. This indicates that FBLN7 can affect the phenotypic transformation of VSMCs. The transition of VSMCs from a contractile phenotype to a synthetic phenotype plays a crucial role in vascular remodeling 7. To validate these findings at the protein level, western blot analysis showed a substantial reduction in contractile markers in the aortas of Ang II-infused FBLN7^+/+^ mice, while the absence of FBLN7 mitigated this downregulation **(Figure 4C)**. Conversely, specific overexpression of FBLN7 in mice further suppressed the expression of these proteins **(Figure S3B-E)**. Immunohistochemical and immunofluorescence staining of aortic sections confirmed these findings, demonstrating that FBLN7 deficiency partially rescued the Ang II-mediated downregulation of α-SMA, SM22α, and CNN1, while FBLN7 overexpression exacerbated their decline **(Figure 4D, Figure S3F-I)**. These results underscore the role of FBLN7 in promoting the Ang II-induced phenotypic transition of VSMCs to a synthetic state.

To further validate the role of FBLN7 in VSMCs phenotypic switching, we conducted *in vitro* experiments in VSMCs. Western blot analysis showed that in VSMCs treated with control adenovirus (adNC), Ang II stimulation led to an increase in FBLN7 expression and a decrease in α-SMA, CNN1, and SM22α levels **(Figure 4E-I)**. When VSMCs were transfected with adenovirus overexpressing FBLN7 (adFBLN7), these effects were amplified **(Figure 4E-I)**. Conversely, FBLN7^-/-^ VSMCs showed a less reduction in contractile markers upon Ang II treatment compared to FBLN7^+/+^ cells **(Figure S3J-M)**. Notably, during this phenotypic transition, VSMCs underwent morphological alterations, transitioning from an elongated, spindle-like shape to a more rounded form. Immunocytochemical staining for α-SMA, CNN1, and SM22α showed that Ang II stimulation triggered this transition, characterized by the loss of specific markers and morphological alterations. Modulating FBLN7 expression through overexpression strategies was found to exacerbate these phenotypic changes **(Figure 4J)**. Collectively, these findings underscore the pivotal role of FBLN7 in regulating the phenotypic transition of VSMCs.

### FBLN7 inhibits smooth muscle contraction gene expression by modulating the ROCK/MRTF-A signaling pathway

**Figure 5:**
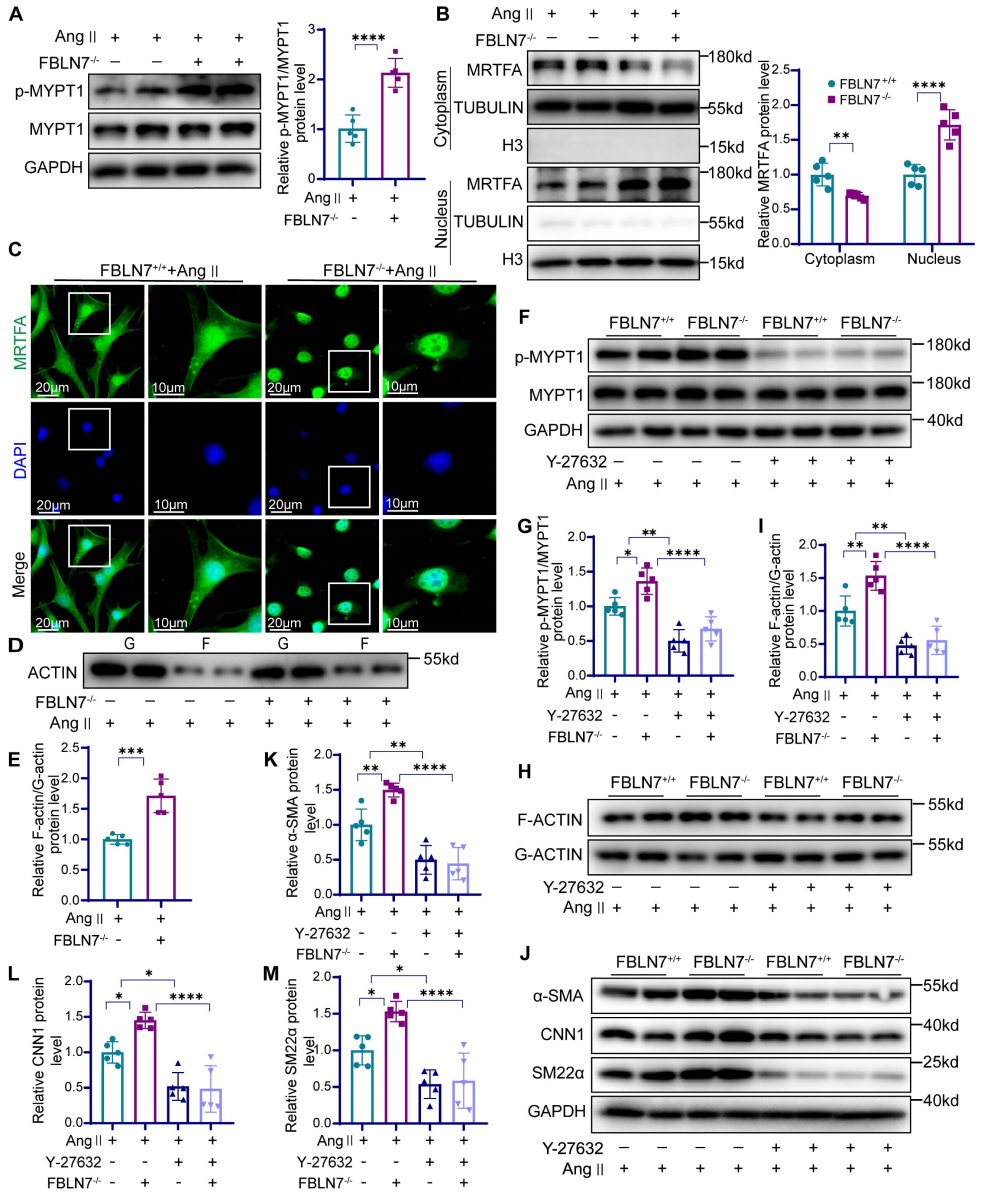
** The absence of FBLN7 can facilitate ROCK activation and nuclear translocation of MRTF-A.** (A) Western blot analysis and the relative quantification of p-MYPT1 and MYPT1 expression in Ang II-treated FBLN7^-/-^ and FBLN7^+/+^ VSMCs (n = 5). (B) Western blot analysis and the relative quantification of nuclear and cytoplasmic expression of MRTF-A in Ang II-treated FBLN7^-/-^ and FBLN7^+/+^ VSMCs (n = 5). (C) Cellular immunofluorescence analysis of nuclear and cytoplasmic expression of MRTF-A in Ang II-treated FBLN7^-/-^ and FBLN7^+/+^ VSMCs. (D-E) Western blot analysis and the relative quantification of G-actin and F-actin expression in Ang II-treated FBLN7^-/-^ and FBLN7^+/+^ VSMCs (n = 5). (F-G) Western blot analysis and the relative quantification of p-MYPT1 and MYPT1 in VSMCs from FBLN7^+/+^ and FBLN7^-/-^ mice treated with Ang II in the presence or absence of Y-27632 (10 μmol/L) (n = 5). (H-I) Western blot analysis and the relative quantification of G-actin and F-actin in VSMCs from FBLN7^+/+^ and FBLN7^-/-^ mice treated with Ang II in the presence or absence of Y-27632 (10 μmol/L) (n = 5). (J-M) Western blot analysis and the relative quantification of α-SMA, CNN1, and SM22α in VSMCs from FBLN7^+/+^ and FBLN7^-/-^ mice treated with Ang II in the presence or absence of Y-27632 (10 μmol/L) (n = 5). Data are presented as mean ± SEM. Statistical significance was assessed using unpaired two-tailed Student's t-test (A, E), one-way (G, I, K-M) or two-way (B) ANOVA followed by Bonferroni post hoc analysis. *p < 0.05, **p < 0.01, ***p < 0.001 and ****p < 0.0001.

The ras homolog family member A (RhoA)/ROCK signaling pathway plays a crucial role in regulating VSMC differentiation through serum response factor (SRF)-dependent transcription 26. While previous studies have demonstrated that FBLN7 can inhibit RhoA activation in endothelial cells 27, its interaction with ROCK in VSMCs remains unclear. Next, we detected the phosphorylation state of myosin phosphatase target subunit 1 (p-MYPT1), a well-characterized substrate of ROCK. Phosphorylation at Thr-696 serves as a marker of ROCK activation in VSMCs 28, 29. Our findings revealed that FBLN7 absence led to elevated phosphorylated MYPT1 levels **(Figure 5A)**, whereas its overexpression correlated with diminished pMYPT1 levels **(Figure S4A)**. MRTF-A is a key nuclear transcription factor in the ROCK-regulated smooth muscle phenotype pathway, typically binding to G-actin in the cytoplasm. When released from G-actin, MRTF-A translocates to the nucleus, where it activates VSMC-specific promoters, thereby modulating the contractile phenotype. We examined the subcellular distribution of MRTF-A following FBLN7 intervention and observed that FBLN7^-/-^ VSMCs exhibited increased nuclear MRTF-A levels, coupled with reduced cytoplasmic levels **(Figure 5B)**. Immunofluorescence confirmed these results, showing enhanced nuclear accumulation in FBLN7 deficiency VSMCs **(Figure 5C)**, whereas FBLN7 overexpressing decreased nuclear MRTF-A fluorescence. **(Figure S4B)**. As MRTF-A nuclear translocation prompts G-actin polymerization into F-actin, we also evaluated the F-actin/G-actin ratio. FBLN7^-/-^ VSMCs displayed a high ratio, suggesting that less MRTF-A remained bound to G-actin, thus prompting its conversion to F-actin **(Figure 5D-E)**. Conversely, FBLN7 overexpression resulted the opposite trend **(Figure S4C)**.

To further investigate whether FBLN7 operates through this pathway, we used the ROCK inhibitor Y-27632. In Ang II-treated FBLN7^-/-^ VSMCs, elevated levels of p-MYPT1 and an increased F-actin/G-actin ratio were observed, both of which were reduced upon Y-27632 treatment **(Figure 5F-I)**. Western blot analysis demonstrating that the enhanced nuclear translocation of MRTF-A, a hallmark of FBLN7 deficiency in VSMCs, was reversed by Y-27632 **(Figure S4D)**. Furthermore, Y-27632 treatment inhibited the phenotypic reversion of smooth muscle contraction in FBLN7^-/-^ VSMCs **(Figure 5J-M)**. The G protein-coupled receptor agonist sphingosine-1-phosphate (S1P) can activate the phosphorylation of the downstream target effector molecule ROCK by activating RhoA, functioning as an agonist of ROCK. Upon S1P treatment, we observed increased p-MYPT1 levels and enhanced nuclear MRTF-A translocation. Interestingly, in FBLN7-overexpressing cells, S1P treatment reversed the FBLN7-induced decreases in p-MYPT1 and nuclear MRTF-A levels **(Figure S5)**. In summary, these findings suggest that FBLN7 inhibits MRTF-A nuclear translocation through the ROCK/MRTF-A pathway, suppressing the transcription of genes related to the smooth muscle contractile phenotype. This ultimately contributes to the phenotypic transition of VSMCs from a contractile to a synthetic state.

### FBLN7 binds to SDC4 and inhibits its dimerization

**Figure 6:**
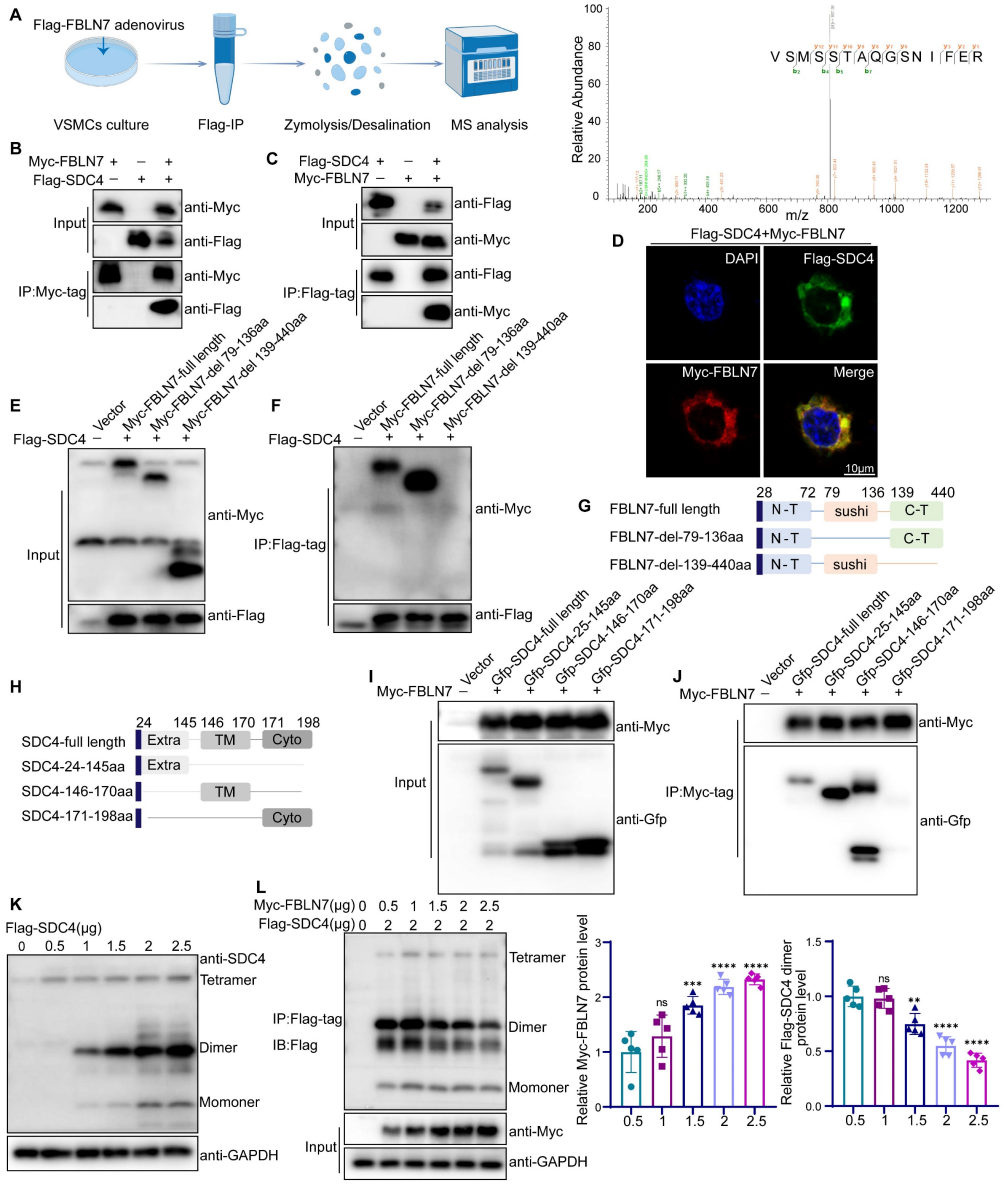
** FBLN7 binds to SDC4 and affects the formation of SDC4 dimers.** (A) A schematic representation of the quantitative proteomic screening for proteins that bind to FBLN7 (Left), along with an MS/MS spectrum displaying the peptide VSMSSTAQGSNIFER from SDC4 (Right). (B-C) Co-immunoprecipitation and immunoblotting analyses of the interactions between Myc (FBLN7) and Flag (SDC4) in HEK293T. (D) Representative confocal images showing co-localization of Myc (FBLN7) and Flag (SDC4). (E-F, I-J) Myc-tagged FBLN7 truncations and GFP-tagged SDC4 truncations were transfected into HEK293T cells, immunoprecipitation and immunoblotting analyses were performed on cell lysates. (G-H) Schematic diagram of FBLN7 and SDC4 mutant structures. (K) Immunoblotting analysis of SDC4 tetramer, dimer and monomer positions. (L) Co-immunoprecipitation analysis and the relative quantification of the effects of FBLN7 on SDC4 dimer formation (n = 5). Data are presented as mean ± SEM. Statistical significance was assessed using one-way ANOVA followed by Bonferroni post hoc analysis, ns indicates no significant difference, **p < 0.01, ***p < 0.001 and ****p < 0.0001.

To further explore the molecular mechanisms by which FBLN7 affects vascular remodeling, we investigated its potential receptors. HSPGs are known receptors of FBLN7 15. SDC4, a member of the HSPGs family, is widely expressed in vascular tissues 30, and mediate the phenotypic transformation of VSMCs via the RhoA pathway 31, 32. We conducted LC-MS/MS analysis using VSMCs transfected with Flag-FBLN7 adenovirus, and the mass spectrometry data confirmed SDC4 as a binding protein of FBLN7 **(Figure 6A, Table S4)**. We employed co-immunoprecipitation (Co-IP) to evaluate the interaction between FBLN7 and SDC4 in VSMCs **(Figure S6A)**. We then confirmed this interaction in HEK293T cells. Overexpression plasmids for Myc-tagged FBLN7 and Flag-tagged SDC4 were constructed and co-transfected into HEK293T cells. After 24 hours, we extracted the cell proteins and performed immunoprecipitation using an anti-Myc antibody, followed by detection with an anti-Flag antibody. Western blot analysis revealed a significant interaction between Myc-FBLN7 and Flag-SDC4** (Figure 6B)**. Similarly, immunoprecipitation using an anti-Flag antibody and detection with an anti-Myc antibody confirmed this interaction **(Figure 6C)**. Dual immunofluorescence staining and confocal microscopy further demonstrated co-localization of the two proteins **(Figure 6D)**. These findings indicate that FBLN7 can bind to SDC4.

Next, we identified the specific domains of FBLN7 and SDC4 responsible for their interaction. Studies have shown that the FBLN7-C region (residues 139-440), encompassing the EGF domain and the C-terminal segment, plays a crucial functional role. To determine if this region or the sushi domain (residues 79-136) mediates the interaction, we constructed truncated variants of FBLN7 (FBLN7-delete-79-136aa, FBLN7-delete-139-440aa) **(Figure 6G)**. Co-IP analysis revealed that deletion of the 139-440aa region disrupted the FBLN7-SDC4 interaction, while deletion of the 79-136aa region did not, indicating that the 139-440aa region is crucial for SDC4 binding **(Figure 6E-F)**. We also explored the functional domains of SDC4, which include the extracellular domain (SDC4-24-145aa, Extra), the transmembrane domain (SDC4-146-170aa, TM), and the cytoplasmic domain (SDC4-171-198aa, Cyto) **(Figure 6H)**. Co-IP analysis showed that both the extracellular (24-145aa) and transmembrane (146-170aa) domains of SDC4 bind to FBLN7 **(Figure 6I-J)**.

The SDC4 extracellular domain mediates interactions with ligands, while the transmembrane domain is essential for SDC4 homodimerization. This sparked our investigation into whether FBLN7 influences the dimerization of SDC4. Our findings reveal that overexpression of FBLN7 in VSMCs significantly reduced the expression of SDC4 dimers **(Figure S6B)**. This observation was further confirmed in HEK293T cells. By transfecting HEK293T cells with varying concentrations of SDC4 plasmids, we identified the molecular weight of SDC4 dimers** (Figure 6K)**, consistent with previous studies 33. When we co-transfected increasing amounts of FBLN7 plasmids with a fixed amount of SDC4, Co-IP analysis showed that as FBLN7 expression increased, the dimeric form of SDC4 progressively decreased **(Figure 6L)**.

### Molecular dynamics (MD) simulation reveals allosteric regulation of FBLN7 and SDC4 transmembrane segment

**Figure 7:**
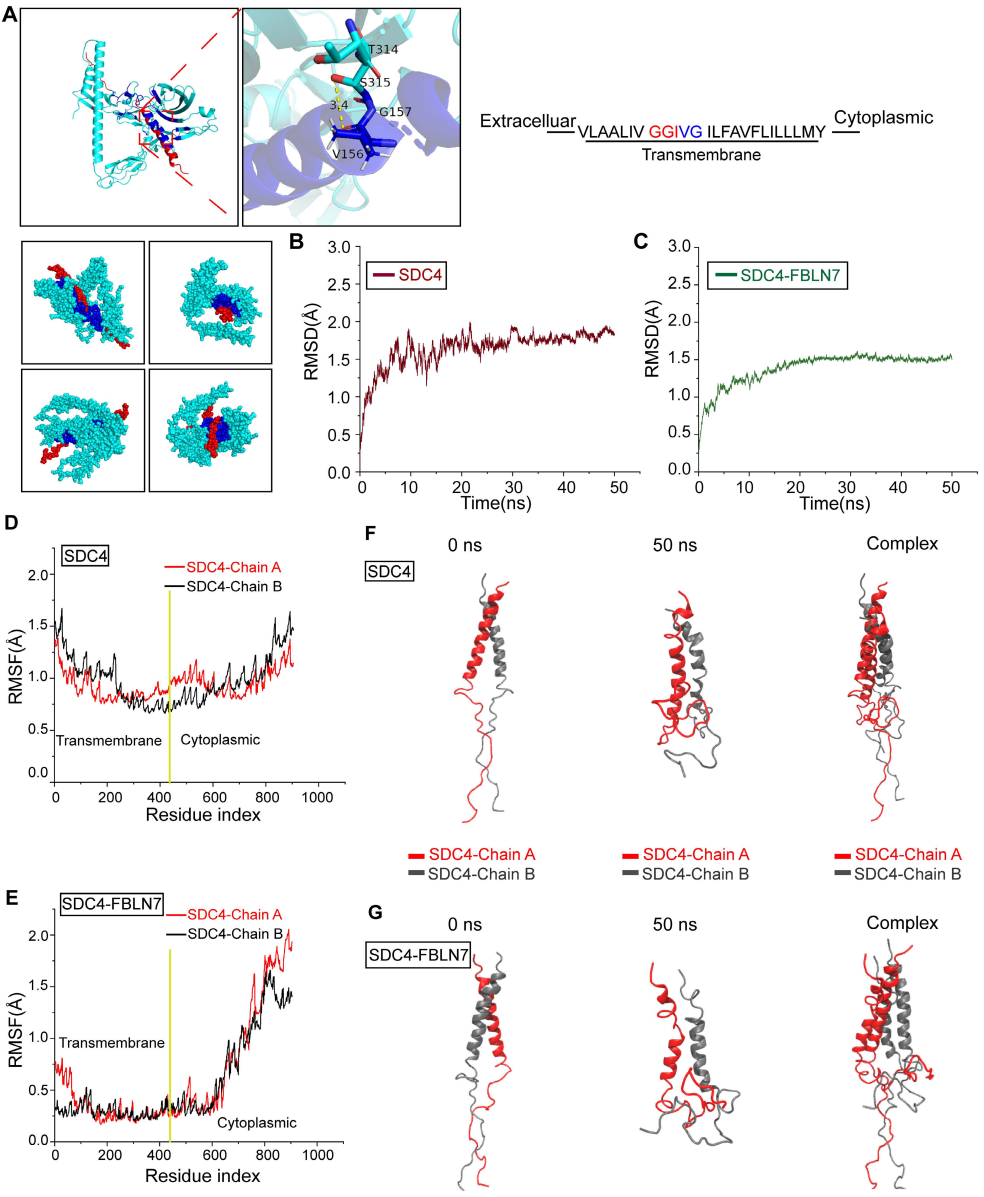
** FBLN7 binds to the GGIVG motif of SDC4 and dissociates SDC4 dimers.** (A) Schematic diagram of molecular docking between FBLN7 and SDC4, along with a magnified view of the binding sites (green, FBLN7; red, SDC4; blue, binding site). (B) Root mean square deviation (RMSD) changes during the simulation process of SDC4 without FBLN7 binding. (C) RMSD changes during the simulation process of SDC4 complex bound to FBLN7. (D) Root mean square fluctuation (RMSF) changes during the simulation process of SDC4 without FBLN7 binding. (E) RMSF changes during the simulation process of SDC4 complex bound to FBLN7. (F) Conformational changes in the two peptide chains of SDC4 at the beginning and end of the simulation without FBLN7 binding. (G) Conformational changes in the two peptide chains of SDC4 at the beginning and end of the simulation with FBLN7 binding.

To further explore how FBLN7 influences SDC4 dimer formation and potential allosteric effects, we conducted MD simulations. The GGIVG motif in the transmembrane segment of SDC4 is a crucial site for dimerization, as previously reported 34. Molecular docking indicated the binding between FBLN7 and this GGIVG motif, suggesting that this interaction may represent a crucial site where FBLN7 influences the dimerization of SDC4 **(Figure 7A)**. Subsequently, we performed 50-nanoseconds (ns) MD simulations on SDC4 dimer with and without FBLN7 binding. Root mean square deviation (RMSD) analysis revealed that both systems stabilized after 10 ns, indicating the stable existence of the two systems **(Figure 7B-C)**. Root mean square fluctuation (RMSF) was used to compare the flexibility of SDC4 amino acid residues before and after FBLN7 binding. The transmembrane segment of SDC4 exhibited a significant decrease in flexibility after binding with FBLN7, while the cytoplasmic segment remained largely unaffected, suggesting a tighter interaction between FBLN7 and the transmembrane region of SDC4 **(Figure 7D-E)**. We then compared the conformational changes of both systems throughout the simulation. In the absence of FBLN7 binding, the SDC4 dimer remained in a stable binding state throughout the 50 ns simulation **(Figure 7F)**. However, after FBLN7 binding, the two chains of the SDC4 dimer gradually dissociated **(Figure 7G)**. These results suggest that FBLN7 binds tightly to SDC4 and disrupts its dimer formation. While studies have shown that SDC4 is related to the phenotypic transformation of VSMCs, the role of SDC4 dimerization in this process has not yet been explored.

### FBLN7 and SDC4 interact to regulate the phenotypic switching of VSMCs

**Figure 8:**
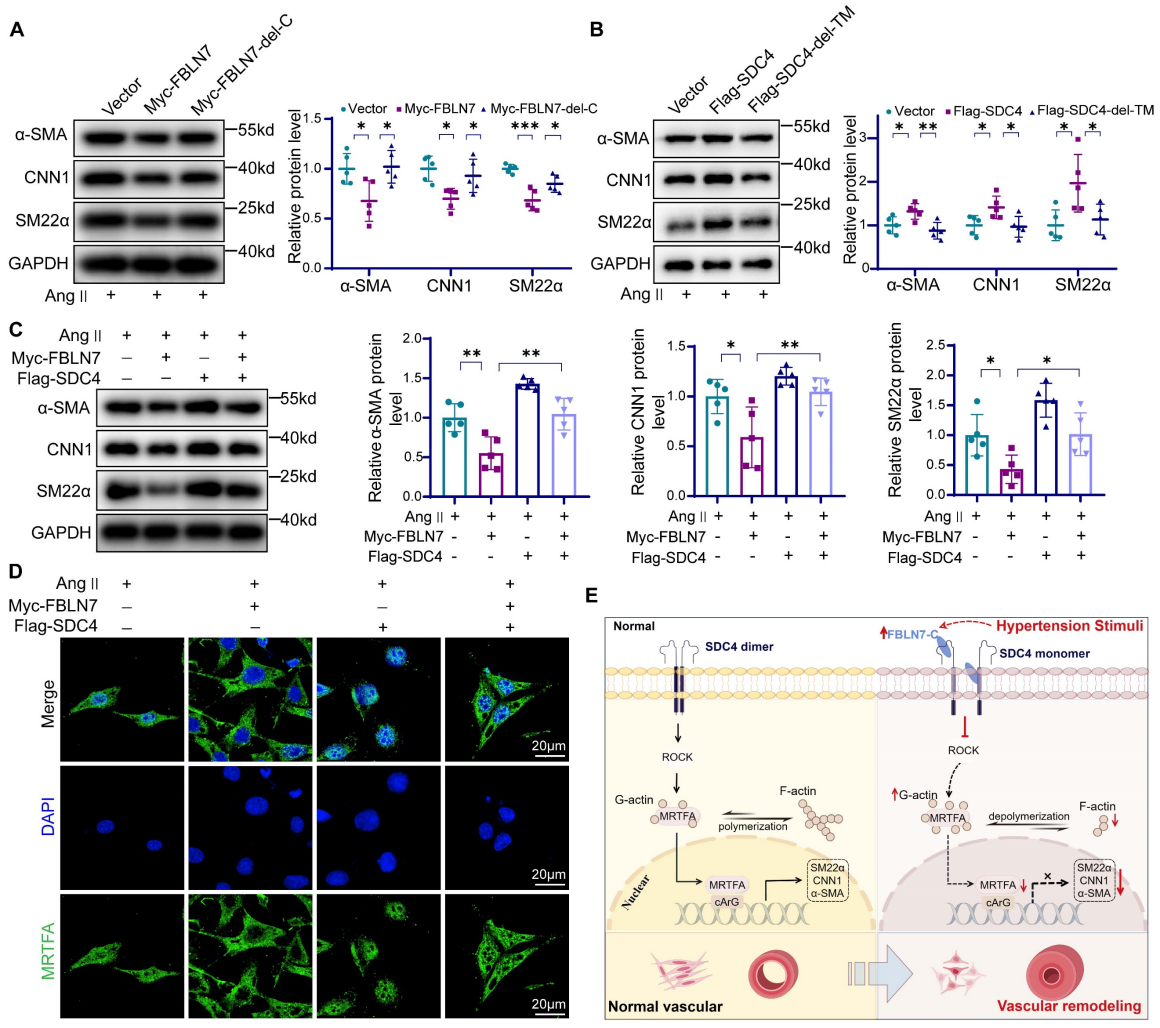
** Effects of FBLN7-SDC4 interaction on phenotypic transformation of VSMCs.** (A) Western blot analysis and the relative quantification of α-SMA, CNN1, and SM22α in VSMCs transfected with FBLN7 full-length and FBLN7-delete-139-440aa (FBLN7-del-C) truncated fragments (n = 5). (B) Western blot analysis and the relative quantification of SM22α, α-SMA, and CNN1 in VSMCs transfected with SDC4 full-length and SDC4-delete-146-170aa (SDC4-del-TM) truncated fragments (n = 5). (C) Western blot analysis and the relative quantification of SM22α, α-SMA, and CNN1 in VSMCs transfected with Flag-SDC4 or Myc-FBLN7 plasmids (n = 5). (D) Immunofluorescence analysis of the nuclear-cytoplasmic distribution of MRTF-A in VSMCs transfected with Flag-SDC4 or Myc-FBLN7 plasmids. (E) Graphical abstract. Data are presented as mean ± SEM. Statistical significance was assessed using one-way ANOVA followed by Bonferroni post hoc analysis. *p < 0.05, **p < 0.01, ***p < 0.001.

We next examined the effect of the FBLN7-SDC4 interaction in VSMCs phenotypic transition. To start, we investigated the function of the FBLN7-C by overexpressing the FBLN7-delete-139-440aa (FBLN7-del-C) mutant and the full-length FBLN7 in VSMCs. The results indicated that the FBLN7-139-440aa deletion mutant retained the expression of contractile markers in VSMCs **(Figure 8A)**. Then we transfected VSMCs with full-length SDC4, the SDC4-delete-146-170aa (SDC4-del-TM). Full-length SDC4 enhanced the expression of contractile genes in VSMCs, while the deletion of the 146-170aa transmembrane domain abolished this effect** (Figure 8B)**, indicating that the transmembrane domain 146-170aa is essential for smooth muscle phenotypic transition. Finally, we conducted a rescue experiment *in vitro*, which showed that SDC4 could partially restore the expression of VSMCs contractile markers reduced by FBLN7 overexpression** (Figure 8C)**. SDC4 also counteracted the downregulation of p-MYPT1 levels caused by FBLN7 overexpression **(Figure S7)**. Immunofluorescence and western blot results show that SDC4 can alleviate the reduction of MRTF-A nuclear entry induced by FBLN7 **(Figure 8D, Figure S7)**. These results suggest that SDC4 mediates the vascular smooth muscle phenotypic transformation induced by FBLN7.

## Discussion

In this study, we demonstrate the critical role of FBLN7 in vascular remodeling. Our findings reveal that FBLN7 primarily participates in vascular remodeling by regulating genes associated with VSMCs contractile function and cytoskeletal rearrangement. Moreover, we identified that the interaction between FBLN7 and cell membrane receptors is a key pathway for its functional execution. Specifically, the interaction between FBLN7 and SDC4 leads to the inhibition of SDC4 function and the dissociation of its homodimers. This suppresses the ROCK/MRTF-A pathway, resulting in reduced expression of genes related to the smooth muscle contractile phenotype.

The fibulin family is known to play an important role in elastic fibers and basement membranes 35. However, our research reveals a distinct function for FBLN7 in vascular pathology. Unlike other fibulins implicated in vascular elasticity 36-40, the vascular tissues of FBLN7 knockout mice did not exhibit pathological vascular changes, which emphasizes its unique role. Our observations in clinical and animal vascular remodeling models show elevated levels of FBLN7 in serum and aortic tissues, supporting its potential as a biomarker for disease progression. Moreover, *in vivo* and *in vitro* experiments demonstrate that FBLN7 regulates VSMCs phenotype switching, revealing its biological function within VSMCs.

Several studies have highlighted the importance of the FBLN7-C domain (135-440 aa) as a functional region responsible for immune inflammation, adhesion, and migration activities 19, 21, 27. Our research also found that FBLN7-C is a crucial functional domain for regulating smooth muscle cell phenotype switching and a key domain for interacting with cell membrane receptors. Recombinant FBLN7-C binds to HUVECs, inducing the clustering of integrins and other adhesion molecules at adhesion sites, and persistently activating focal adhesion kinase, p130Cas, and Ras-related C3 botulinum toxin substrate 1 (Rac1) while inhibiting RhoA activation 22. Although the exact mechanism by which FBLN7-C disrupts the balance between Rac1 and RhoA is unclear, it likely prevents RhoA activation by binding to several cell surface receptors. ROCK, the primary downstream effector of RhoA 26, 41, promotes the phosphorylation of MYPT1 and myosin light chain upon activation 42, facilitating VSMC contraction, actin polymerization, and stress fiber formation 43. In our study, we found that overexpression of FBLN7 reduced MYPT1 phosphorylation, indicating its ability to block ROCK activation.

Numerous studies have shown that the SRF cofactor MRTF-A regulates the expression of smooth muscle cell differentiation markers 44, 45. ROCK-mediated actin polymerization stimulates specific gene transcription in VSMCs by regulating the nuclear localization of MRTF-A 29. Abnormalities in the MRTF-A pathway have been reported to result in the downregulation of key contractile markers, such as α-SMA, CNN1, and smooth muscle myosin heavy chain, thus impacting VSMC contractility 32. MRTF-A binds to G-actin in the cytoplasm 46, and when G-actin polymerizes into F-actin, MRTF-A dissociates and enters the nucleus, forming a ternary complex with SRF and the CArG box to activate transcription of VSMC-specific promoters 47. Our study demonstrated that FBLN7 influences MRTF-A distribution between the nucleus and cytoplasm, as well as the F-actin/G-actin ratio, indicating that FBLN7 modulates smooth muscle contractile-related genes through the ROCK/MRTF-A pathway. These conclusions are further supported by the use of the ROCK inhibitor Y-27632 and the agonist S1P 48.

HSPGs are known receptors for FBLN7. Syndecan, a transmembrane heparan sulfate proteoglycan, has four members. Studies have shown that Syndecan-4 is the only member of the family that is ubiquitously expressed in nucleated cells and plays a critical role in cell adhesion, migration, and signal transduction 30, 49. Research has shown that SDC4 modulates cellular mechanics in response to local tension by activating the kindlin-integrin-RhoA pathway, unveiling a novel function of SDC4 in cellular mechanics 50. Furthermore, silencing SDC4 has been shown to enhance abdominal aortic aneurysm formation and VSMC phenotypic switching through the RhoA/G-actin/MRTF-A pathway 32, providing evidence that SDC4 can regulate ROCK function and participate in vascular remodeling. The function of SDC4 in our study aligns with these previous findings. Additionally, our experiments demonstrated the interaction between FBLN7-C and SDC4, as well as the structural domains involved. We discovered that FBLN7 binds to the transmembrane domain of SDC4, sparking our research interest. Under normal physiological conditions, SDC4 typically forms dimers or higher-order oligomers, which are crucial for its stability, trafficking, and signaling functions 34, 51, 52. The GGIVG motif in the transmembrane domain of SDC4 is a key site for homodimerization. After binding with FBLN7, we observed a marked reduction in SDC4 dimerization. We then performed MD simulations to examine the conformational changes of SDC4 upon interaction with FBLN7, and the results showed that FBLN7 binds to the GGIVG motif of SDC4 and affects SDC4 dimer aggregation. By constructing truncations lacking the transmembrane domain, we confirmed that SDC4 dimerization plays a role in smooth muscle phenotypic transformation. These findings enrich the understanding of SDC4's function in smooth muscle cells.

Our experiments have some limitations. On one hand, we did not conduct experiments using VSMCs-specific FBLN7 knockout or transgenic mice, which would have provided more direct evidence for the function of FBLN7 in VSMCs. However, we utilized adenovirus-mediated VSMCs-specific overexpression of FBLN7 in an effort to partially address this limitation. On the other hand, since no direct inhibitors or agonists of SDC4 dimerization are currently available, there is no direct evidence proving that the effects of FBLN7 on downstream pathways are mediated through SDC4 dimer formation. Further studies are needed to explore the role of SDC4 in smooth muscle phenotypic transformation and the interaction between FBLN7 and HSPGs.

In conclusion, these results clearly indicate that FBLN7 involved in the pathogenesis of hypertension-induced vascular remodeling. The interaction between FBLN7 and cell membrane receptors plays a key role in the occurrence of vascular remodeling. These findings reveal a specific link between FBLN7 and vascular remodeling, providing a new target for clinical drug research.

## Methods

### Generation of FBLN7- knockout mice

FBLN7 knockout mice (FBLN7^-/-^) were produced by Saiye Biotechnology (Guangzhou, China) by injecting Cas9 and gRNA into the fertilized eggs of C57BL/6J mice using CRISPR/Cas9 strategy. FBLN7^-/-^ mice were obtained by mating FBLN7^+/-^ mice. Genomic DNA of mouse tail was extracted, amplified and sequenced using corresponding primers, which were as follows: Forward 5'-AAGACATAAACATCACCTTTGGC-3', and Reverse 5'-CACATTGCTTGCATTTGTG-3' to verify the genotype of the knockout mice. FBLN7^-/-^ mice did not exhibit overt developmental or health defects. 10-week-old male FBLN7^-/-^ mice were randomly selected for modeling.

### Ang II-induced vascular remodeling in mice

Ten-week-old wide type (FBLN7^+/+^) or FBLN7^-/-^ mice were selected as experimental subjects. An osmotic mini-pump (Alzet model 2004, Cupertino, USA) was used to administer Ang II (A9525, Sigma, Missouri, USA) or saline at a rate of 490 ng/kg/min for 28 days to establish a vascular remodeling model (as described previously) 5, 53. Mice were anesthetized with isoflurane before inserting the micro-osmotic pump. Sham-operated mice were implanted with osmotic mini-pumps containing 0.9% saline. The systolic blood pressure (SBP) of the mice was measured weekly by non-invasive tail-cuff plethysmography (BP-98A, Softron, Tokyo, Japan). Vascular ultrasound was performed before the start of the experiment and on the 28th day. At the end of the experiment, the mice were euthanized with an overdose of anesthetic to collect tissue.

The experimental animal ethics certification was approved by the Experimental Animal Committee of Qilu Hospital of Shandong University (Jinan, Shandong, China) (Permit Number: DWLL-2022-166).

### *In vivo* VSMC-specific FBLN7 overexpression

The specific overexpression of FBLN7 in mice was achieved using adeno-associated virus-9 (AAV9) provided by GenePharma (Shanghai, China), driven by the SM22α gene promoter to ensure specific manipulation of VSMCs. In the AAV9-SM22α-FBLN7 group, six-weeks-old male C57BL/6J mice were injected via the tail vein with AAV9-FBLN7 (5×10^^11^ vector genomes (vg)/mouse), while the control group received the empty AAV9 vector. Four weeks after mice were injected with AAV9-FBLN7, aortic tissue was obtained to evaluate the overexpression effect of AAV9-FBLN7.

### Study population and blood samples

Thirty-five hypertensive patients and 47 age-matched healthy individuals were recruited from Qilu Hospital of Shandong University for plasma collection. Hypertension was defined as systolic blood pressure ≥ 140 and/or diastolic blood pressure ≥ 90 without the use of antihypertensive drugs, or patients with a history of hypertension who are currently taking antihypertensive drugs 54. This study was approved by the Ethics Committee of Qilu Hospital of Shandong University with the ethics permission number KYLL-2024(ZM)-1128. All patients and their representatives provided written informed consent. This study conformed to the principles of the Helsinki Declaration. Clinical characteristics are presented in the appendix** (Table S1)**.

### Enzyme-linked immunosorbent assay (ELISA)

Peripheral blood supernatants from patients or mice were collected by centrifugation, and the levels of FBLN7 were measured using an ELISA kit according to the manufacturer's instructions (Mmbio, Jiangsu, China).

### Cell culture and reagents

MASMCs were isolated from the thoracic aorta of mice using an enzymatic digestion method, with slight modifications based on a previous protocol 55. Briefly, the aorta tissue above the renal artery bifurcation was dissected and rinsed in cold phosphate-buffered saline (PBS). After removing the fat tissue, the entire vessel was placed in a solution containing 0.2% collagenase type II (B204, Worthington, USA) and incubated at 37 °C in a cell culture incubator for 20 minutes. After 20 minutes, the vessel was removed, and the adventitia was carefully peeled off using fine forceps in a "sock-like" manner. The vessel tissue was then cut into small pieces (1-2 mm) and further digested with 0.1% collagenase type II at 37 °C for 6-8 hours. MASMCs were maintained in Dulbecco's Modified Eagle Medium/Nutrient Mixture F-12 (DMEM/F12)(Cell-Max, Beijing, China) supplemented with 20% fetal bovine serum(FBS) (Cell-Box, Hunan, China) and 1% penicillin/streptomycin, and identified by immunofluorescence staining for α-SMA. Cells from passages 2-4 were used for further experiments. Ang II (1 μmol/L) (A9525, Sigma, Missouri, USA), Y-27632 (10 μmol/L) (HY-10071, MCE, Shanghai, China) and S1P (10 μmol/L) (HY-108496, MCE, Shanghai, China) were used in cell treatment.

### Construction and transfection of plasmid and adenovirus

The plasmids provided by GenePharma (Shanghai, China). Standard PCR techniques were employed to amplify the full-length cDNA sequences of mouse SDC4 and FBLN7, which were then subcloned into the pcDNA 3.1 vector, appending a C-terminal Flag tag to SDC4 and a C-terminal MYC tag to FBLN7. Through PCR, expression plasmids were constructed for EGFP-tagged full-length SDC4 and its truncated variants, cloned into the pcDNA3.1-EGFP vector. Similarly, the expression plasmid for Myc-labeled full-length FBLN7 and its truncated mutants was created by PCR and cloned into the pcDNA3.1-Myc vector. For comparison, pcDNA 3.1 vectors carrying Flag, Myc, and EGFP tags served as control plasmids. According to the manufacturer's instructions, the plasmids were transfected into VSMCs or HEK293T cells using Lipofectamine 3000 (Thermo Fisher Scientific, MA, USA).

GeneChem (Shanghai, China) designed recombinant FBLN7 adenoviruses and empty vectors carrying the Flag-tagged sequence. VSMCs were incubated with adFBLN7 and adenovirus negative control at a multiplicity of infection (MOI) of 100 PFU/cell for 12 hours.

### RNA-seq analysis

Total RNA was collected from FBLN7^+/+^ VSMCs and FBLN7^-/-^ VSMCs after treatment with Ang II. The RNA sequencing was conducted by Novogene (Beijing, China). Differentially expressed genes (DEGs) with fold changes > 1.5 and adjusted p-values < 0.05 were considered significant. Kyoto Encyclopedia of Genes and Genomes (KEGG) and Gene Ontology (GO) enrichment analyses were performed using the Novogene free cloud platform.

### Liquid chromatography-tandem mass spectrometry (LC-MS/MS) analysis

Flag antibody was added to VSMCs for conducting Immunoprecipitation (IP) experiments. The LC-MS/MS analysis was performed by GeneChem (Shanghai, China). Based on the scores and masses of the detected proteins, we conducted a screening process to identify substrate proteins capable of binding to FBLN7. The secondary mass spectrum in the article was exported by pLabel software 56.

### Western blot analysis

Cells and aorta tissues were collected. Using RIPA lysis buffer (Solarbio, Beijing, China) containing 1% PMSF (Solarbio, Beijing, China) and 1% protease inhibitor cocktail (Cwbio, Beijing, China), cells or tissues were lysed on ice and then centrifuged at 12,000 rpm for 15 minutes to collect the protein. Protein concentration was measured using a Pierce BCA Protein Assay Kit (Cwbio, Beijing, China). Samples were mixed with 5× loading buffer (Cwbio, Beijing, China), denatured in a 99 ℃ metal bath for 10 minutes, and then used for protein electrophoresis. For the extraction of nuclear and cytoplasmic proteins, a nuclear-cytoplasmic protein extraction kit is employed, following the steps provided in the kit's instructions (P0027, Beyotime, Beijing, China). When the purpose of the western blot experiment is to detect protein dimers, a non-reducing loading buffer (Epizyme, Shanghai, China) is used for elution. Samples (20-30 μg protein) were separated by SDS-PAGE and transferred to PVDF membranes (Millipore, Massachusetts, USA) by wet transfer. After blocking with 5% milk for 1-2 hours, the membranes were incubated with the corresponding primary antibodies at 4 ℃ overnight. Then, they were incubated with HRP-conjugated secondary antibodies (Zsbio, Beijing, China) at room temperature for 1-2 hours. Immunoreactive bands were visualized with Amersham Imager 680 (GE, USA) or Tanon 5200 (Tanon, China), the blots were quantified with densitometry by Image J. The specific information about the antibodies used in this study can be found in the appendix **(Table S5)**.

### Real-time Quantitative RT-PCR (PCR)

Total RNA was extracted from mouse aorta using RNA extraction kit (Vazyme, Nanjing, China). Then, RNA samples were reverse transcribed into cDNA using a reverse transcription kit (Vazyme, Nanjing, China). Finally, quantitative analysis was conducted through real-time fluorescent quantitative polymerase chain reaction on a real-time PCR detection system using SYBR Green PCR Master Mix (Vazyme, Nanjing, China), following the manufacturer's protocol. The primers used for RT-PCR are shown in the appendix** (Table S6)**.

### Co-immunoprecipitation assay (Co-IP)

After washing with ice-cold PBS, cells were lysed with cell lysis buffer for IP (P0013, Beyotime, Beijing, China) containing 1% PMSF and the cell lysates were pre-incubated with target antibodies overnight at 4 °C, and then incubated with protein A/G magnetic beads (HY-K0202, MCE, Shanghai, China) for 2 hours. The magnetic beads were washed three times with 0.5% Tween-20 in PBS, and the precipitated proteins were eluted from the magnetic beads by resuspending in 1 × SDS PAGE loading buffer and boiling at 95 °C for 5 minutes. Protein analysis was performed by western blot, and the special secondary antibody avoiding light and heavy chains is used (ab131366, Abcam, Cambridge, England).

### Histological analysis

Aortic tissues were immediately fixed in 4% paraformaldehyde after isolation. After 24 hours of fixation, they were rinsed in running water for 5 hours to remove excess paraformaldehyde. The tissues were then dehydrated using an automated tissue processor and embedded in paraffin. Paraffin sections of 5 micrometers in thickness were stained with Masson's trichrome (G1346, Solarbio, Beijing, China), H&E, and Verhoeff's van Gieson (G1598, Solarbio, Beijing, China) according to the manufacturer's instructions. For immunohistochemistry, an HRP/DAB detection kit (ZSGB-Bio, Beijing, China) was employed. Briefly, aortic sections were deparaffinized in xylene and rehydrated through graded ethanol. Antigen retrieval was performed according to the user manual. Endogenous peroxidase activity was quenched with 3% H2O2 for 15 minutes, and sections were blocked with 5% bovine serum albumin (BSA) at room temperature for 1 hour. Diluted primary antibodies were added and incubated overnight, followed by incubation with appropriate HRP-conjugated secondary antibodies at room temperature for 30 minutes. The color was developed using the kit's chromogenic solution under a microscope. Finally, sections were counterstained with hematoxylin, dehydrated, and sealed. Image J software was used for staining evaluation and quantification.

### Immunofluorescence assay

Immunofluorescence staining of aortic tissue was performed using paraffin sections of mouse thoracic aorta. Initially, the tissue was dewaxed to water, followed by antigen retrieval by microwave heating. The tissue was sealed with 5% BSA at room temperature for 1 hour, and then incubated overnight at 4 °C with diluted primary antibodies. For immunofluorescence staining in cells, VSMCs were seeded on 24-well plate cell slides, and subsequent experimental treatments were conducted after cell adhesion. Following treatment, cells were fixed with 4% paraformaldehyde for 15 minutes, permeabilized with 0.5% Triton X-100 for 10 minutes, blocked with 5% BSA for 1 hour, and then incubated overnight at 4 °C with the corresponding primary antibodies. Subsequently, the cells were incubated with fluorescent secondary antibodies at room temperature for 1 hour. The fluorescent fixing medium containing DAPI (ab104139, Abcam, Cambridge, England) was used to stain the cell nuclei. Fluorescent signals were detected using a confocal laser scanning microscope (LSM710, Carl Zeiss, Germany) or a fluorescence microscope (Nikon, Tokyo, Japan).

### Determination of the F-/G-Actin ratio in VSMCs

The F-/G-Actin extraction method refers to the previous protocol 57. VSMCs were lysed on ice for 10 minutes using RIPA buffer followed by centrifugation at 15,000 ×g for 30 minutes. Soluble G-actin was collected in the supernatant, while insoluble F-actin remained in the precipitate. The precipitate was washed three times with PBS and then resuspended in lysis buffer (1.5 mM guanidine hydrochloride, 1 mM sodium acetate, 1 mM CaCl2, 1 mM ATP, and 20 mM Tris-HCl; pH 7.5) (Aladdin, Shanghai, China) and incubated on ice for 1 hour. The samples were vortexed every 15 minutes on a rotator to convert F-actin into soluble G-actin. After centrifugation at 15,000 ×g for 30 minutes, the supernatant containing the F-actin was collected. Western blot analysis was performed on samples using an actin-specific antibody (ab230169, Abcam, Cambridge, England).

### Molecular docking and molecular dynamics simulation

The initial structure of FBLN7 (AF-Q53RD9-F1) was predicted by AlphaFold 58, the initial structure of the transmembrane and cytosolic segments of SDC4 originates from the article by Vesa P. Hytönen *et al.* 50. The FBLN7 protein was attached to the potential binding site of the SDC4 protein using the ZDOCK 3.0.2 program, and the conformation with the highest score was selected based on the ZDOCK score 59. Image analysis was performed using Pymol.

The MD simulations were conducted using the GROMACS 2018.4 software package under constant temperature and pressure conditions 60, along with periodic boundary conditions. The simulations employed the all-atom OPLS-AA force field in conjunction with the TIP3P water model 61. All bonds involving hydrogen atoms were constrained using the LINCS algorithm, while electrostatic interactions were computed via the PME method 62, 63. The simulation temperature was maintained at 310 K through the V-rescale temperature coupling scheme, and pressure was controlled at 1 bar with the Parrinello-Rahman method. Prior to the main simulations, an energy minimization of the system was performed using the steepest descent algorithm over 50,000 steps. Subsequently, 1 ns of NVT and NPT equilibration simulations were carried out to achieve the final equilibrium stage. These conditions were adopted for all subsequent simulations. An integration time step of 2 fs was utilized for all simulations. Following this, a 50 ns equilibrated MD simulation was executed, with conformations saved every 10 ps. The simulation results were analyzed using GROMACS' built-in tools and visualized with the VMD software.

### Statistical analysis

Statistical analyses were performed using GraphPad Prism 9.0 (GraphPad Software, La Jolla, California). All data are presented as mean ± SEM. The normality of the data was assessed using the Shapiro-Wilk test. Statistical significance between two groups was analyzed using an unpaired Student's t-test. When more than two groups were involved, one-way or two-way ANOVA with subsequent Bonferroni post-hoc testing was used to analyze differences between groups. A p-value less than 0.05 was considered statistically significant. All biological experiments were repeated at least five times using independent cell cultures or individual animals.

## Supplementary Material

Supplementary figures and tables.

## Abbreviations

Ang II: angiotensin II
AAV: adeno-associated virus
AAA: abdominal aortic aneurysm
CNN1: calponin1
Co-IP: co-immunoprecipitation
FBLN7: fibulin7
GO: gene ontology
H3: histone H3
HSPGs: heparan sulfate proteoglycans
KEGG: kyoto encyclopedia of genes and genomes
LC-MS/MS: liquid chromatography-tandem mass spectrometry
MRTF-A: myocardin-related transcription factor A
MYPT1: myosin phosphatase target subunit 1
MAECs: mouse aortic endothelial cells
MAFs: mouse aortic fibroblasts
MPMs: mouse peritoneal macrophages
ROCK: rho-associated protein kinase
RMSF: root mean square fluctuation
RMSD: root mean square deviation
RhoA: ras homolog family member A
SDC4: syndecan-4
SM22α: smooth muscle 22α
α-SMA: α-smooth muscle actin
VSMCs: vascular smooth muscle cells
WB: western blot
WT: wild type
