# Supplementary material for: FBLN7 mediates vascular smooth muscle cell phenotype switching and vascular remodeling in hypertension
Source: Theranostics. 2024 Nov 4;14(19):7569–88. doi: 10.7150/thno.102593 (PMC11626941; doi:10.7150/thno.102593)
Supplement: Supplementary file 1 — Supplementary figures and tables. [file thnov14p7569s1.pdf]

Supplementary Materials for

FBLN7 mediates vascular smooth muscle cell phenotype switching and vascular remodeling in hypertension

Guoqing Yao, Xuehui Zheng, Yang Hu, Yuan Zhao, Binghui Kong, Yun Ti*, Pei li Bu*

State Key Laboratory for Innovation and Transformation of Luobing Theory; Key Laboratory of Cardiovascular Remodeling and Function Research, Chinese Ministry of Education, Chinese National Health Commission and Chinese Academy of Medical Sciences; Department of Cardiology, Qilu Hospital of Shandong University, Jinan, 250012, China

***Corresponding author:** Pei li Bu, MD, PhD, and Yun Ti, MD, PhD State Key Laboratory for Innovation and Transformation of Luobing Theory; Key Laboratory of Cardiovascular Remodeling and Function Research, Chinese Ministry of Education, Chinese National Health Commission and Chinese Academy of Medical Sciences; Department of Cardiology, Qilu Hospital of Shandong University, Jinan, 250012, China.

E-mail: bupeili@sdu.edu.cn; tiyun0820@163.com

This PDF file includes:

1. Supplemental Figure 1-7
2. Supplemental Table 1-6

Supplemental Figures

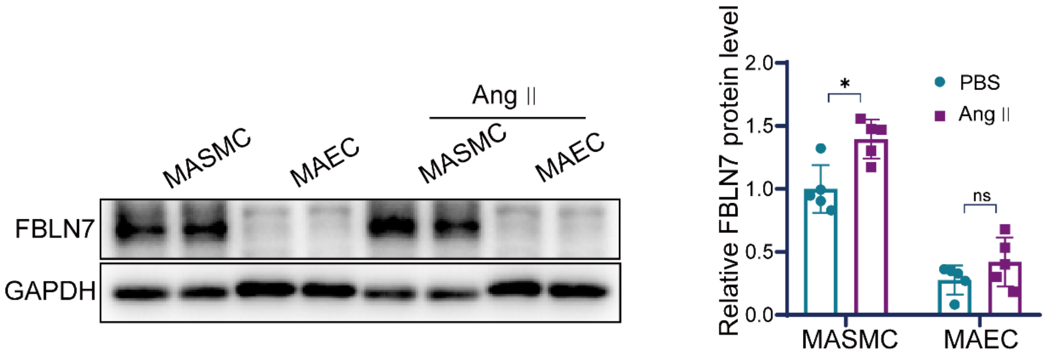

Figure S1. The expression of FBLN7 in MASMCs increases after Ang II stimulation. Western blot analysis and the relative quantification of FBLN7 in MASMCs and MAECs treated with PBS or Ang II. Data are presented as mean ± SEM. Statistical significance was assessed using two-way ANOVA with Bonferroni post hoc analysis. *$p < 0.05$, ns indicates no significant difference.

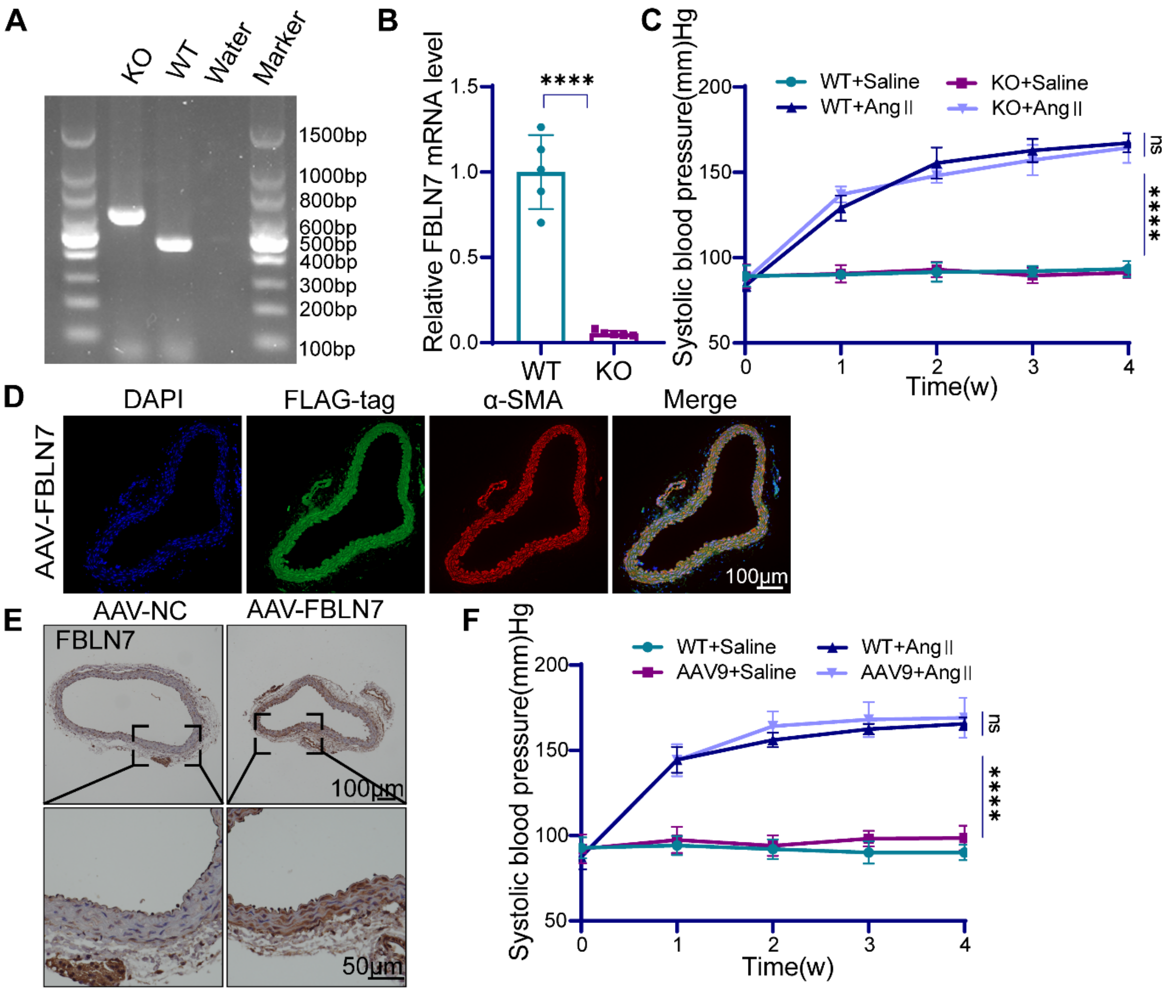

Figure S2. FBLN7 deletion or overexpression did not affect systolic blood pressure in Ang II-induced mice. (A) Verification of the FBLN7$^{-/-}$ genotype by PCR. WT, wild-type mice; KO, FBLN7$^{-/-}$ mice. (B) Changes in FBLN7 mRNA levels in the aortas of WT and KO mice were quantified using RT-qPCR (n = 5). (C) Systolic blood pressure measurements were recorded in mouse experiments (n = 5). (D) Immunofluorescence staining of aortic tissue from mice injected with AAV9-SM22α-FBLN7 carrying the Flag tag after 4 weeks. FLAG-tag (green), α-SMA (red, marking smooth muscle cells), and DAPI (blue, marking cell nuclei) were used. (E) Immunohistochemical staining of FBLN7 in aorta of mice injected with AAV9-SM22α-FBLN7 (AAV-FBLN7) or empty vector (AAV-NC) for 4 weeks. (F) Systolic blood pressure was recorded in mouse experiments (n = 5). Data are presented as mean ± SEM. Statistical significance was assessed using unpaired two-tailed Student's t-test (B) or two-way ANOVA with Bonferroni post hoc analysis (C, F). ****$p < 0.0001$, ns indicates no significant difference.

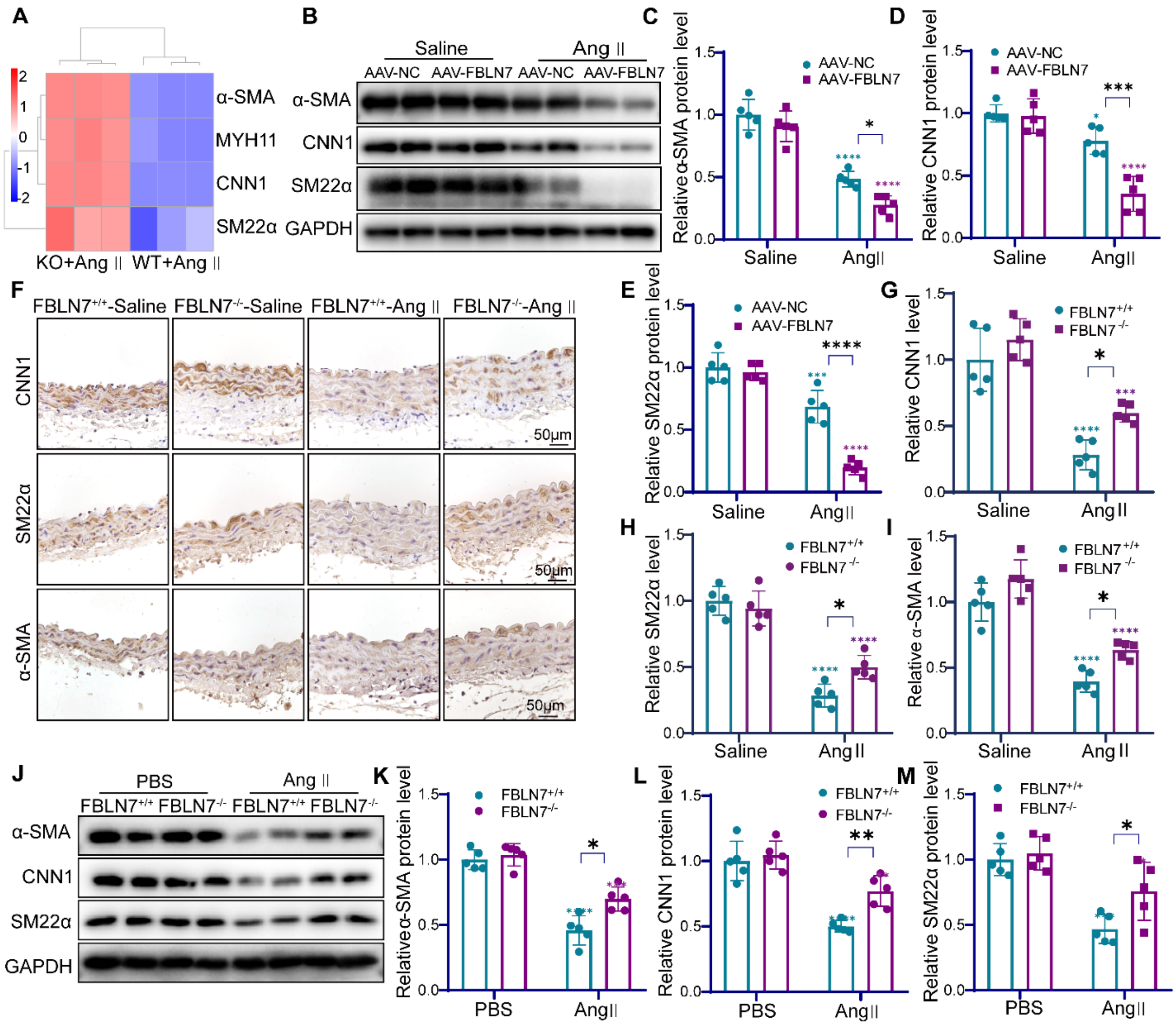

Figure S3. FBLN7 modulates the phenotypic transformation of VSMCs. (A) Heatmap of the VSMC phenotypic transition marker (α-SMA, CNN1, SM22α and MYH11) (n = 3). (B-E) Western blot analysis and the relative quantification of α-SMA, CNN1, and SM22α expression in aorta tissue of AAV9-SM22α-FBLN7 or empty AAV9 vector mice after infusion of saline or Ang II for 4 weeks (n = 5). (F-I) Representative images of α-SMA, CNN1, and SM22α immunohistochemical staining and the relative quantification in aorta tissues of FBLN7$^{+/+}$ and FBLN7$^{-/-}$ mice after infusion of saline or Ang II for 4 weeks (n = 5). (J-M) Western blot analysis and the relative quantification of α-SMA, CNN1, and SM22α in FBLN7$^{-/-}$ and FBLN7$^{+/+}$ VSMCs treated with PBS or Ang II (n = 5). Data are presented as mean ± SEM. Statistical significance was assessed using two-way ANOVA with Bonferroni post hoc analysis. *$p < 0.05$, **$p < 0.01$, ***$p < 0.001$ and ****$p < 0.0001$.

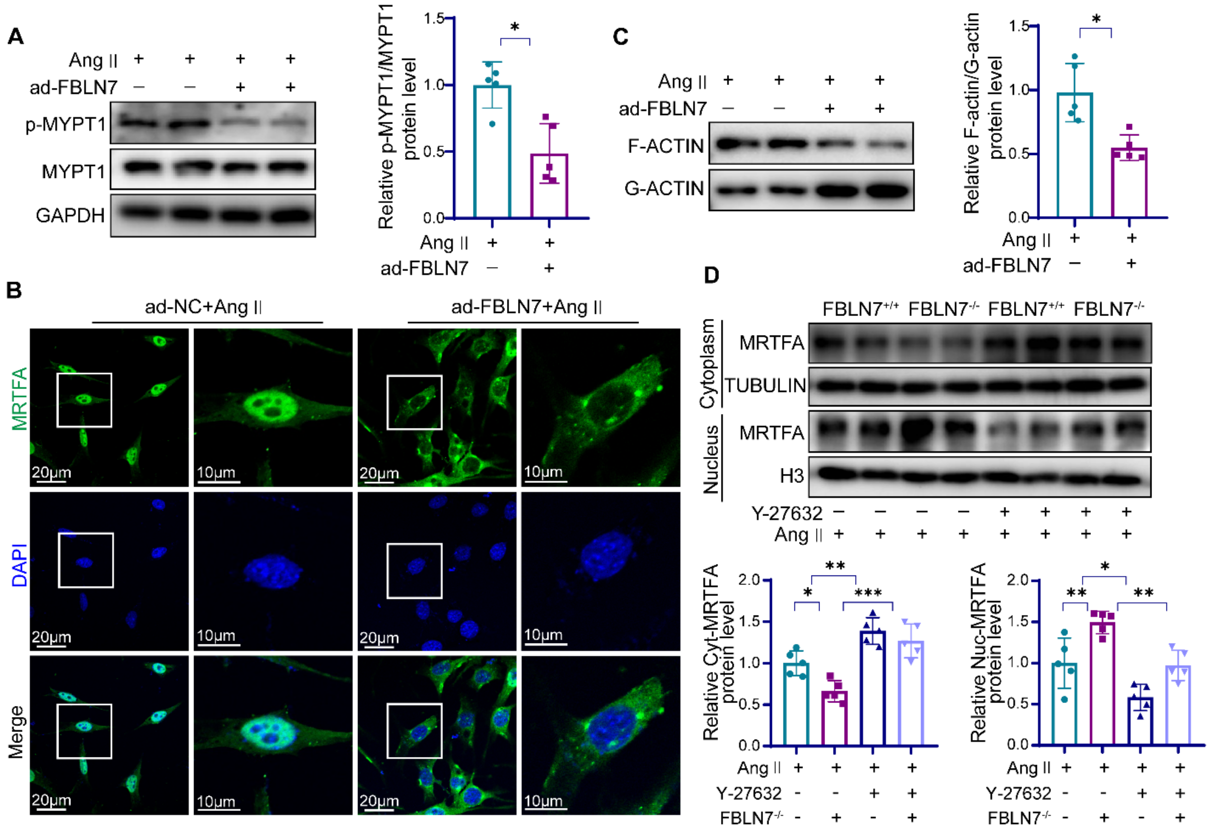

Figure S4. Overexpression of FBLN7 inhibits ROCK activation and nuclear translocation of MRTF-A. (A) Western blot analysis and the relative quantitative of p-MYPT1 and MYPT1 in adNC and adFBLN7 VSMCs treated with Ang II (n = 5). (B) Immunofluorescence was used to show the nuclear and cytoplasmic distribution of MRTFA in adNC and adFBLN7 VSMCs treated with Ang II (n = 5). (C) Western blot analysis and the relative quantitative of F-actin, G-actin in adNC and adFBLN7 VSMCs treated with Ang II (n = 5). (D) FBLN7$^{-/-}$ and FBLN7$^{+/+}$ VSMCs were treated with Ang II in the presence or absence of Y-27632 (10 μmol/L). Western blot analysis and the relative quantification were performed for MRTF-A, TUBULIN, and H3 (n = 5). Data are presented as mean ± SEM. Statistical significance was assessed using unpaired two-tailed Student's t-test (A, C) or one-way ANOVA followed by Bonferroni post hoc analysis (D). *$p < 0.05$, **$p < 0.01$, ***$p < 0.001$.

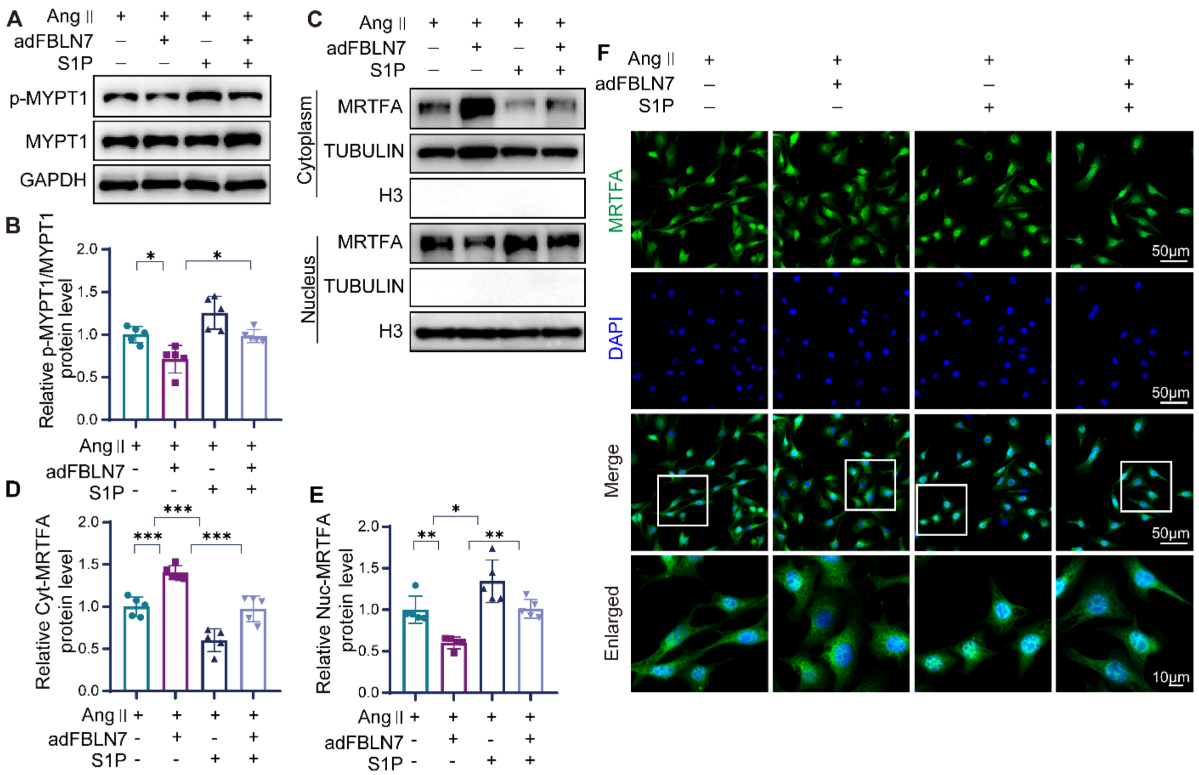

Figure S5. S1P treatment reversed the reduction in p-MYPT1 and nuclear MRTF-A levels induced by overexpression of FBLN7. (A-B) Western blot analysis and the relative quantification of p-MYPT1, MYPT1 in adNC or adFBLN7 VSMCs treated with Ang II in the presence or absence of S1P (10 μmol/L) ($n = 5$). (C-E) Western blot analysis and the relative quantification of nuclear and cytoplasmic expression of MRTF-A in adNC or adFBLN7 VSMCs treated with Ang II in the presence or absence of S1P (10 μmol/L) ($n = 5$). (F) Cellular immunofluorescence analysis of nuclear and cytoplasmic expression of MRTF-A in adNC or adFBLN7 VSMCs treated with Ang II in the presence or absence of S1P (10 μmol/L). Data are presented as mean ± SEM. Statistical significance was assessed using one-way ANOVA followed by Bonferroni post hoc analysis. *$p < 0.05$, **$p < 0.01$, ***$p < 0.001$.

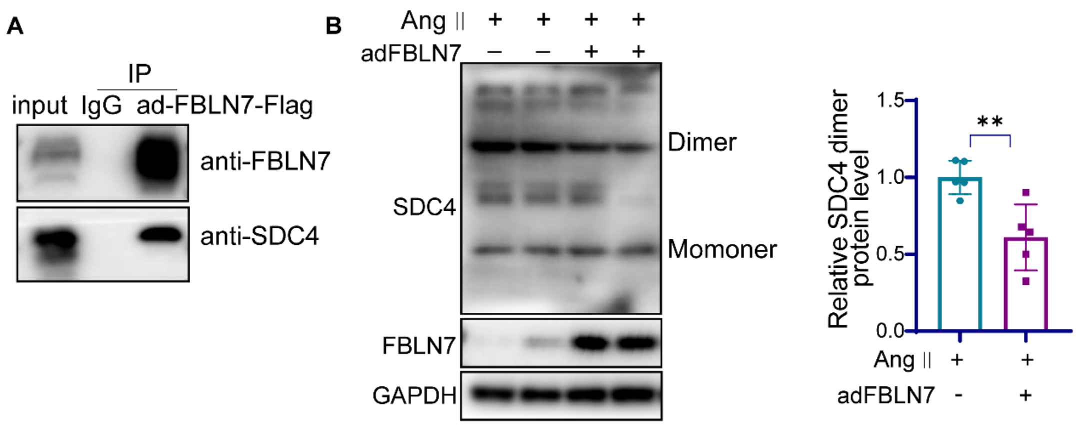

Figure S6. FBLN7 binds to SDC4 and inhibits its dimerization in VSMCs. (A) In mouse VSMCs, an adenoviral vector overexpressing FBLN7 fused with a Flag tag was introduced. Cellular proteins were then extracted after 24 h, and an immunoprecipitation assay was performed using an anti-Flag antibody. Subsequently, the immunoprecipitated proteins were detected with SDC4 and FBLN7 antibody. (B) FBLN7 was overexpressed in mouse VSMCs, the results revealed a significant reduction in the dimeric form of SDC4 following the overexpression of FBLN7 ($n = 5$). Data are presented as mean ± SEM. Statistical significance was assessed using unpaired two-tailed Student's t-test. **$p < 0.01$.

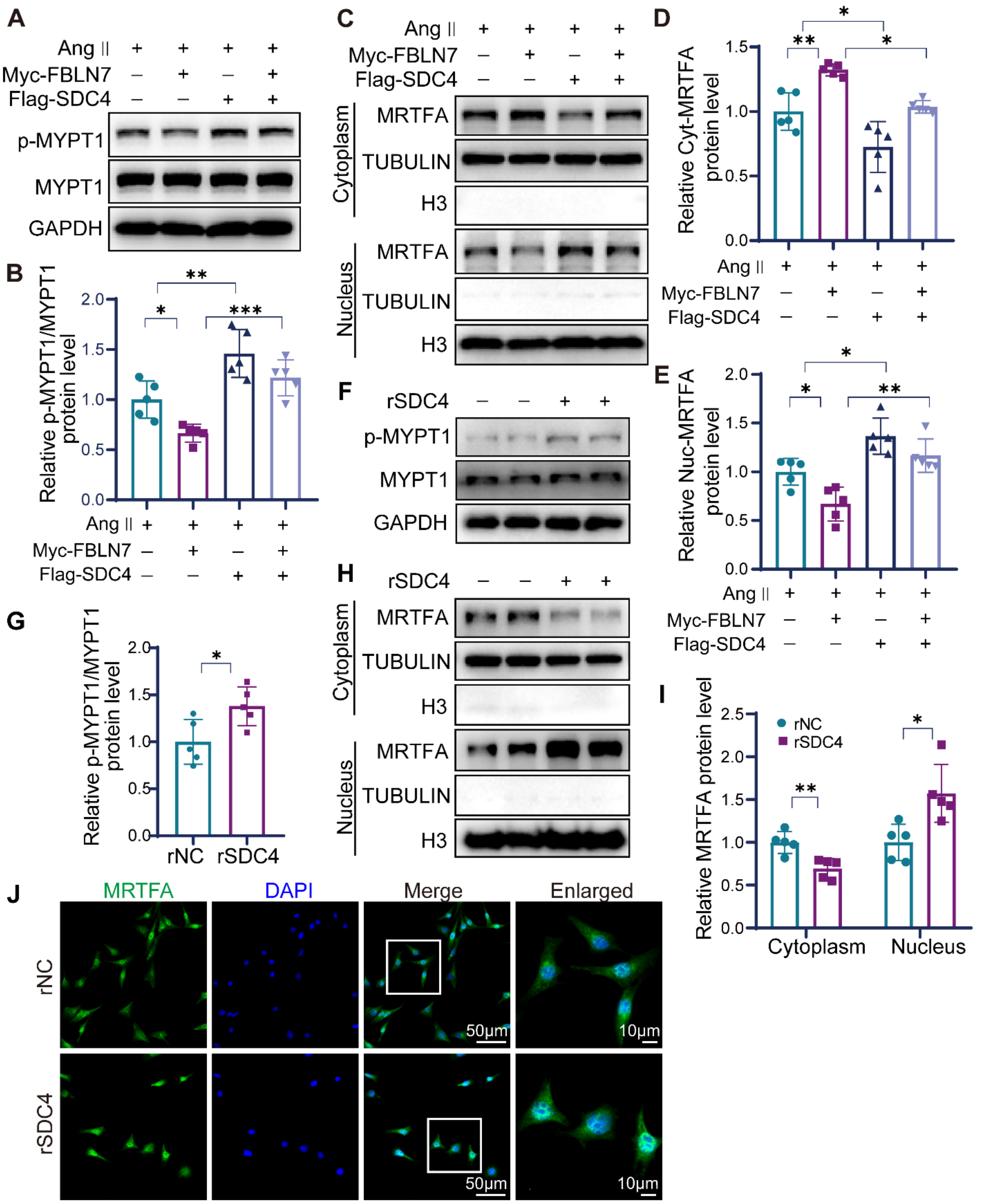

Figure S7. SDC4 facilitates the activation of the ROCK/MRTF-A pathway. (A-B) Western blot analysis and the relative quantification of p-MYPT1, MYPT1 in VSMCs transfected with Flag-SDC4 or Myc-FBLN7 plasmids (n = 5). (C-E) Western blot analysis and the relative quantification of the nuclear and cytoplasmic expression of MRTF-A in VSMCs transfected with Flag-SDC4 or Myc-FBLN7 plasmids (n = 5). (F-G) Western blot analysis and the relative

quantification of p-MYPT1, MYPT1 in VSMCs treated with recombinant mouse SDC4 protein (rSDC4, 2 μg/mL, HY-P73424, MCE) or rNC ($n = 5$). (H-I) Western blot analysis and the relative quantification of the nuclear and cytoplasmic expression of MRTF-A in VSMCs treated with rSDC4 or rNC ($n = 5$). (J) Immunofluorescence was used to show the nuclear and cytoplasmic distribution of MRTF-A in VSMCs treated with rSDC4 or rNC. Data are presented as mean ± SEM. Statistical significance was assessed using unpaired two-tailed Student's t-test (G); one-way ANOVA (B, D, E) or two-way ANOVA (I) followed by Bonferroni post hoc analysis. *$p < 0.05$, **$p < 0.01$, ***$p < 0.001$.

Supplemental Tables

Table S1. Basic characteristics of patients with essential hypertension and health controls

**Characteristics**	**Control (n = 47)**	**Hypertension (n = 35)**
Male (%)	26 (55.3%)	16 (45.7%)
Age (year)	55.38 ± 9.735	58.4 ± 9.552
Systolic blood pressure (mmHg)	120.7 ± 10.26	144.9 ± 14.82 *
Diastolic blood pressure (mmHg)	73.47 ± 8.243	85.29 ± 13.13 *

*P＜0.05

Table S2. The information of KEGG pathway enrichment

KEGG ID	Description	GeneRatio	padj
mmu04514	cell adhesion molecules	0.043	2.50E-08
mmu03030	DNA replication	0.020	4.48E-08
mmu04151	PI3K-Akt signaling pathway	0.078	1.21E-07
mmu04015	Rap1 signaling pathway	0.055	1.25E-07
mmu04060	cytokine-cytokine receptor interaction	0.051	8.43E-07
mmu04512	ECM-receptor interaction	0.026	3.18E-06
mmu04668	TNF signaling pathway	0.033	3.75E-06
mmu04010	MAPK signaling pathway	0.065	5.51E-06
mmu04020	calcium signaling pathway	0.053	1.11E-05
mmu04110	cell cycle	0.046	1.52E-05
mmu04510	focal adhesion	0.046	0.000282
mmu04062	chemokine signaling pathway	0.038	0.000321
mmu04061	viral protein interaction with cytokine and cytokine receptor	0.019	0.00043
mmu04657	IL-17 signaling pathway	0.025	0.000441
mmu05323	rheumatoid arthritis	0.021	0.000571
mmu04810	regulation of actin cytoskeleton	0.046	0.001063
mmu04270	vascular smooth muscle contraction	0.030	0.001704
mmu04310	Wnt signaling pathway	0.037	0.001704
mmu04022	cGMP-PKG signaling pathway	0.037	0.001878
mmu04080	neuroactive ligand-receptor interaction	0.044	0.002192

Table S3. The information of GO molecular function enrichment

GO ID	Description	GeneRatio	padj
GO:0030545	receptor regulator activity	0.049	1.50E-14
GO:0048018	receptor ligand activity	0.046	1.50E-14
GO:0008083	growth factor activity	0.020	8.72E-09
GO:0019838	growth factor binding	0.022	3.35E-08
GO:0005125	cytokine activity	0.022	1.53E-07
GO:0005126	cytokine receptor binding	0.029	4.60E-06
GO:0005539	glycosaminoglycan binding	0.023	6.04E-06
GO:0004714	transmembrane receptor protein tyrosine kinase activity	0.010	1.91E-05
GO:0008201	heparin binding	0.018	1.91E-05
GO:0019199	transmembrane receptor protein kinase activity	0.012	2.39E-05
GO:0005178	integrin binding	0.015	4.14E-05
GO:0004713	protein tyrosine kinase activity	0.018	4.14E-05
GO:0005520	insulin-like growth factor binding	0.006	7.73E-05
GO:0008009	chemokine activity	0.006	0.000133515
GO:0003779	actin binding	0.038	0.000159424
GO:0017147	Wnt-protein binding	0.006	0.000208889
GO:0005516	calmodulin binding	0.021	0.000428677
GO:0042056	chemoattractant activity	0.006	0.000436222
GO:0004930	G-protein coupled receptor activity	0.031	0.000519897
GO:0004674	protein serine/threonine kinase activity	0.044	0.000703236

Table S4. The sequence of the SDC4-specific peptide and its score identified in mass spectrometry analysis

Sequence	Protein	Score
VSMSSTAQGSNIFER	SDC4	118
APSDVGDDMSNK	SDC4	87
RAPSDVGDDMSNK	SDC4	76
ETEVIDPQDLLEGR	SDC4	72
VSMSSTAQGSNIFER	SDC4	71
RAPSDVGDDMSNK	SDC4	64
KKDEGSYDLGK	SDC4	57
APSDVGDDMSNK	SDC4	51
KDEGSYDLGK	SDC4	47
ELEENEVIPK	SDC4	44
DEGSYDLGK	SDC4	40
DEGSYDLGKKPIYK	SDC4	40
KDEGSYDLGKKPIYK	SDC4	39
ELEENEVIPKR	SDC4	27

Table S5. Antibodies

Target antigen	Vendor or Source	Catalog #	Working concentration
FBLN7	Bioss	bs-13161R	1:100 (IF); 1:200 (IHC)
FBLN7	Novus Biologicals	NBP2-20659	1:2000 (WB)
SM22α	Proteintech	10493-1-AP	1:100 (IF); 1:1000 (WB); 1:200 (IHC)
CNN1	Abcam	ab46794	1:500 (IF); 1:5000 (WB); 1:200 (IHC)
α-SMA	Abcam	ab124964	1:500 (IF); 1:5000 (WB); 1:500 (IHC)
α-SMA	Abcam	ab7818	1:500 (IF)
GAPDH	Proteintech	60004-1-Ig	1:5000 (WB)
TUBULIN	Proteintech	66031-1-Ig	1:10000 (WB)
H3	Abcam	ab1791	1:2000 (WB)
MYPT1	Cell Signaling Technology	2634S	1:1000 (WB)
p-MYPT1	Cell Signaling Technology	5163S	1:1000 (WB)
MRTFA	Proteintech	21166-1-AP	1:100 (IF); 1:1000 (WB)
MRTFA	Abclonal	A8504	1:100 (IF); 1:500 (WB)
GFP-tag	Proteintech	66002-1-Ig	1:50 (CO-IP); 1:2000 (WB)
MYC-tag	Proteintech	60003-2-Ig	1:50 (CO-IP); 1:2000 (WB); 1:200 (IF)
MYC-tag	Proteintech	16286-1-Ig	1:50 (CO-IP); 1:2000 (WB); 1:200 (IF)
FLAG-tag	Thermo Fisher	MA1-91878	1:50 (CO-IP); 1:500 (WB); 1:200 (IF)
SDC4	Immunoway	YT4491	1:500 (WB)
SDC4	Abcam	ab-74139	1:500 (WB)
Actin	Abcam	ab230169	1:2000 (WB)

IF, immunofluorescence; WB, western blot; IHC, immunohistochemical; CO-IP, co-immunoprecipitation assay

Table S6. Quantitative Real-time PCR Primer List

Name	Species	Forward	Reverse
FBLN7	Mouse	GAAGACATCTCCCTTTCAGTGCG	GGCATCCTCAGAAGTCATAGCG
GAPDH	Mouse	AGGTCGGTGTGAACGGATTTG	TGTAGACCATGTAGTTGAGGTCA
